# A Report on Multi-Target Anti-Inflammatory Properties of Phytoconstituents from *Monochoria hastata* (Family: *Pontederiaceae*)

**DOI:** 10.3390/molecules26237397

**Published:** 2021-12-06

**Authors:** Md Mazedul Haq, Md Arifur Rahman Chowdhury, Hilal Tayara, Ibrahim Abdelbaky, Md Shariful Islam, Kil To Chong, Sangyun Jeong

**Affiliations:** 1 Research Center of Bioactive Materials, Department of Bioactive Material Sciences, Division of Life Sciences (Molecular Biology Major), Jeonbuk National University, Jeonju 54896, Korea; haqmazed@jbnu.ac.kr (M.M.H.); 201855347@jbnu.ac.kr (M.A.R.C.); 2 Department of Biochemistry and Molecular Biology, National University, Gazipur 1704, Bangladesh; md-shariful@inceptavaccine.com; 3 Department of Pharmacy, Southeast University, Road 18, Block B, Banani, Dhaka 1213, Bangladesh; 4 School of International Engineering and Science, Jeonbuk National University, Jeonju 54896, Korea; hilaltayara@jbnu.ac.kr; 5 Department of Electronics and Information Engineering, Jeonbuk National University, Jeonju 54896, Korea; ibrahim.abdelbaky@fci.bu.edu.eg; 6 Artificial Intelligence Department, Faculty of Computers and Artificial Intelligence, Benha University, Banha 13518, Egypt

**Keywords:** analgesic activity, chronic inflammation, cytokines storm, drug design, molecular dynamics, admet

## Abstract

This study aims to investigate the potential analgesic properties of the crude extract of *Monochoria hastata (MH)* leaves using in vivo experiments and in silico analysis. The extract, in a dose-dependent manner, exhibited a moderate analgesic property (~54% pain inhibition in acetic acid-induced writhing test), which is significant (** *p* < 0.001) as compared to the control group. The complex inflammatory mechanism involves diverse pathways and they are inter-connected. Therefore, multiple inflammatory modulator proteins were selected as the target for in silico analysis. Computational analysis suggests that all the selected targets had different degrees of interaction with the phytochemicals from the extract. Rutin (RU), protocatechuic acid (PA), vanillic acid (VA), and ferulic acid (FA) could regulate multiple targets with a robust efficiency. None of the compounds showed selectivity to Cyclooxygenase-2 (COX-2). However, regulation of COX and lipoxygenase (LOX) cascade by PA can reduce non-steroidal analgesic drugs (NSAIDs)-related side effects, including asthma. RU showed robust regulation of cytokine-mediated pathways like RAS/MAPK and PI3K/NF-kB by inhibition of EGFR and IKBα (IKK), which may prevent multi-organ failure due to cytokine storm in several microbial infections, for example, SARS-CoV-2. Further investigation, using in vivo and in vitro experiments, can be conducted to develop multi-target anti-inflammatory drugs using the isolated compounds from the extract.

## 1. Introduction

Inflammation is a mechanism of multiple interconnected complex pathways. Diverse synthetic drug compounds are available for the treatment of inflammation. Which often possess intolerable side effects. The toxicity of nonsteroidal anti-inflammatory drugs (NSAIDs) is a major concern for the selection of dosages as a treatment [1,2]. In this regard, multi-target drug design is coined to develop safer NSAIDs [3,4,5]. To address this problem, phytochemicals would be a source for alternative drugs.

*Monochoria hastata (MH)* is an aquatic plant belonging to the water hyacinth family; Pontederiaceae is well recognized in Bangladesh as Boronokha. (English-arrow-Leaf or Pondweed; Hindi: Launkia, Vietnamese: rauMác). Other species of this genus have anti-asthmatic anti-inflammatory, anti-nephrotoxic, analgesic activities, and also exhibit toothache relief action and fever suppressing properties [6,7,8]. Whereas *MH* already has shown antidiarrheals, diuretics, blood cleaning, and anti- Gingivitis activities [9]. Although, the herb is used for different purposes from pain relief to treating long-term inflammations in different region of south Asia and south east Asia [10]. However, there are no scientifically examined reports to confirm these traditional claims.

**Figure 1:**
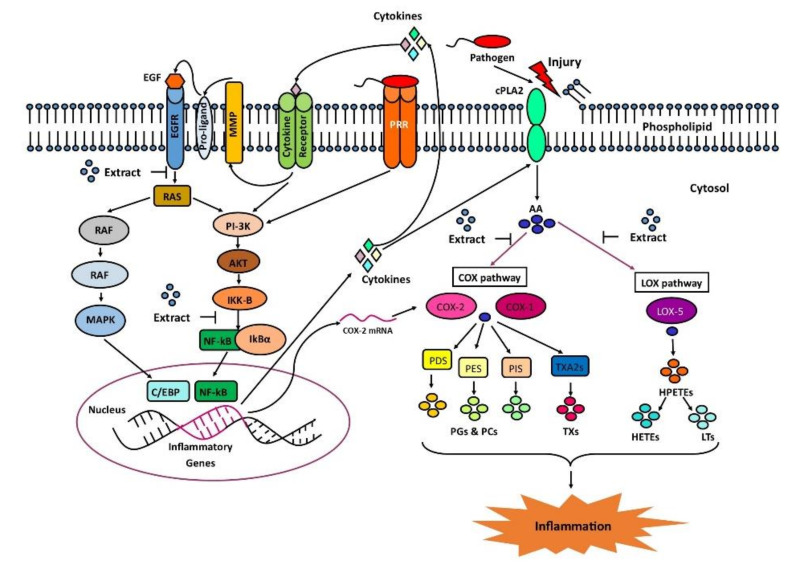
Inflammation can result from injury-mediated conversion of arachidonic acid (AA) metabolites. Synthesis of thromboxanes (TXs, red circles), prostaglandins (PGs, yellow, yellowish-green, and green circles), and leukotrienes (LTs, dark and light sky blue circles) from AA (blue circles) are regulated by largely COX-1 (magenta), COX-2 (pink) and LOX-5 (purple). In multiple cancers, EGFR plays a vital role for COX-2 induced inflammation. Pathogens can bind to PRR and initiate the PI-3k mediated signaling. PRR = pathogen recognition receptor; MMP = *Matrix Metalloproteinase;* EGF(R) = epidermal growth factor (receptor); RAS = rat sarcoma; RAF = rapidly accelerated fibrosarcoma; MAPK = mitogen-activated protein kinase; PI-3K = phosphoinositide 3-kinase; AKT = protein kinase B; C/EBP = CCAAT-enhancer-binding proteins; IKK-B = Inhibitor of nuclear factor kappa-B kinase subunit beta (IKK); NF-kB = nuclear factor kappa-light-chain-enhancer of activated B cells; IkBα = inhibitor of NF-kB alpha; cPLA2 = cytosolic phospholipase A2; P(D,E,I)S = prostaglandin (D,E,I) synthase; TXA2S = thromboxane-A2 synthase; TXs = thromboxanes; HPETEs = hydroperoxyeicosatetraenoic acids; HETEs = hydroxyeicosatetraenoic acids; LT = leukotrienes.

The focus of this study is to evaluate the regulation of some key check points in the complex inflammation pathways using the *MH* crude extract. The canonical inflammatory mechanism is initiated due to any injury to the plasma membrane and consequently activates the cytosolic phospholipase A2 (cPLA2) [11]. The activated cPLA2 initiates a series of reactions that produces arachidonic acid (AA) molecules from membrane phospholipids (Figure 1). As a result, cyclooxygenase (COX) and lipoxygenase (LOX) pathways are turned on. These pathways have a vital role in the pro-inflammatory activities that are tightly associated with fever and pain [12]. Moreover, pathogenic infection can also induce other signaling cascades. Among them, epidermal growth factor receptor (EGFR) is associated with the regulation of pro-inflammatory cytokines and COX-2 expression. Activated EGFR regulates two different downstream pathways [13,14,15,16], which are RAS/MAPK and PI3K/NF-kB pathways. The later pathway can be activated by pathogen recognition receptor (PRR) through pathogenic infection. Dissociation of NF-kB triggers the expression of pro-inflammatory cytokines [17,18,19]. These cytokines are critical because of their ability to reactivate the EGFR and PI3K/NF-kB pathways mediated by cytokine receptors. All these key modulators are considered in this study.

Primarily, writhing test performed in mice model was considered to evaluate the most common traditional claim of analgesic activity of this plant. Therefore, evaluation of analgesic activity is to refute any weak perception and we further develop the study comprising multi-signaling pathways using in silico methods. This helped us to understand the underlying interaction between the inflammatory modulators and the selected compounds.

## 2. Results

### 2.1. Analgesic Activity of Monochoria hastata Leaves Extract

**Figure 2:**
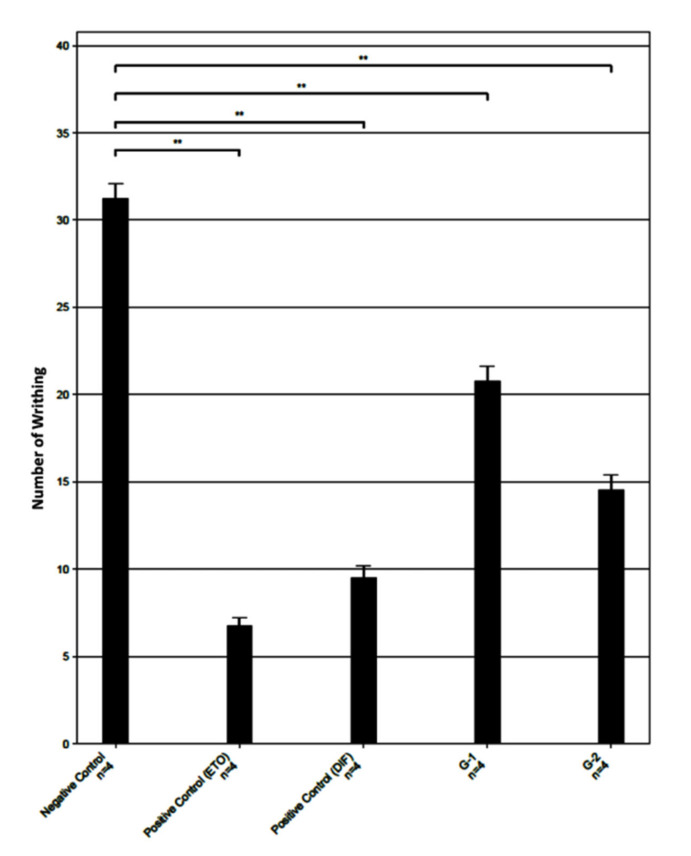
Acetic acid induced writhing response in mice model after treatment with ethanolic extract of *MH* leaves. G-1 is 200 mg/kg and G-2 is 400 mg/kg of *MH* leaves extract. Number of mice in each group, *n* = 4; values are expressed as mean ± S. D.; ** *p* < 0.001 when compared to negative control.

After administering the different doses of ethanolic extract of *MH* leaves with suitable vehicles and standard drug Diclofenac (DIF) and Etoricoxib (ETO) as the positive control, the numbers of writhing reduced and ensured analgesic effects. DIF is able to regulate the both COX-1 and COX-2 non selectively. ETO is included in this study because it can inhibit COX-2 selectively without interfering the COX-1 activity [20]. Therefore, COX-2 selectivity of the selected compounds can be assessed. By comparing the number of writhing with the untreated control group, *MH* showed the reduced writhing response (inhibition of 34% and 54% at the dose of 200 and 400 mg/kg bw, respectively). It is worthwhile to note that standard drugs ETO and DIF exhibited more than 70% inhibition (Table 1). Statistical significance was calculated using analysis of variance (ANOVA), and it was ** *p* < 0.001 for each group in comparison with the saline-treated mice (negative control; Figure 2). These results suggest that *MH* leaves extract shows significant analgesic activity.

### 2.2. In Silico Analysis

Bioactive molecules isolated from plants are thought to execute their mode of action by interacting with proteins or other macromolecules. However, the mechanism of pharmacological responses is poorly characterized due to unspecified targets. Here we performed computational approaches for establishing a linear relationship with the given or claimed pharmacological action. In this study, we used computational docking and molecular dynamics (MD) simulations to inspect the binding modes formed by molecular interactions of previously isolated (reported by Tsun-Thai Chai, et al., 2014) [21] bioactive compounds from *Monochoria hastata* leaves (Table 2). The study was performed against the active sites of COX-1, COX-2, LOX-5, EGFR, and IKK enzymes.

#### 2.2.1. In Silico Prediction of Activity Spectra for Substances (PASS)

Pharmacological activity prediction tool PASS was used to determine the potential biological effects of the isolated compounds. The probable pharmacological functions are listed in Table S1. For the prediction, properties with pharmacological activity (Pa) > 0.7 were selected for reporting. The highest number of pharmacological activities was predicted for protocatechuic acid (PA), followed by syringic acid (SyA) and vanillic acid (VA). All other compounds were also predicted to have a significant number of effects.

#### 2.2.2. Drug-like Properties of Isolated Compounds

The assessment was done for all the isolated compounds to check the drug-likeness properties using the Molinspiration online tool. These properties are important for identifying competitive drug-like molecules and comprehensive drug design [22]. Molecular weight, topological polar surface area (TPSA), miLogP, hydrogen bond donor (HBD), hydrogen bond acceptor (HBA), and the number of rotatable bond (nROTB) (Table 3) were considered to find out the competitiveness of the isolated compounds. It is conceivable that all of them except rutin (RU) follow the rule of five (RO5) proposed by Lipinski, Lombardo, Dominy, and Feeney [23]. Although chlorogenic acid (CA) has six hydrogen bond donors, Lipinski’s violations are not more than one.

#### 2.2.3. Molecular Docking Validation (MDV)

**Figure 3:**
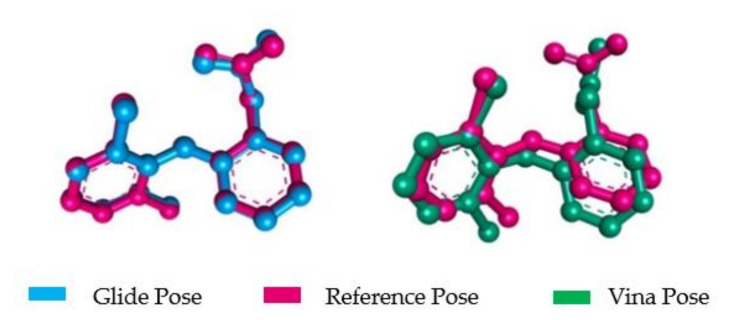
Superimpose of Glide and Vina generated poses over the reference pose.

MDV was carried out for both Glide and Vina by redocking the extracted native ligand (Diclofenac) of COX-2 protein structure (PDB ID: 1PXX). After redocking, both tools produced ligand conformations that were almost identical when superimposed with the native co-crystalized ligand. The best pose showed the docking score of −10.14 and binding affinity −8.9 in glide docking and autodock vina, respectively. For autodock vina the RMSD is 2.275 Å while, for glide docking it is 1.252 Å (Figure 3). Based on this observation, Glide was selected for further docking analysis.

#### 2.2.4. Molecular Docking and Interaction

**Figure 4:**
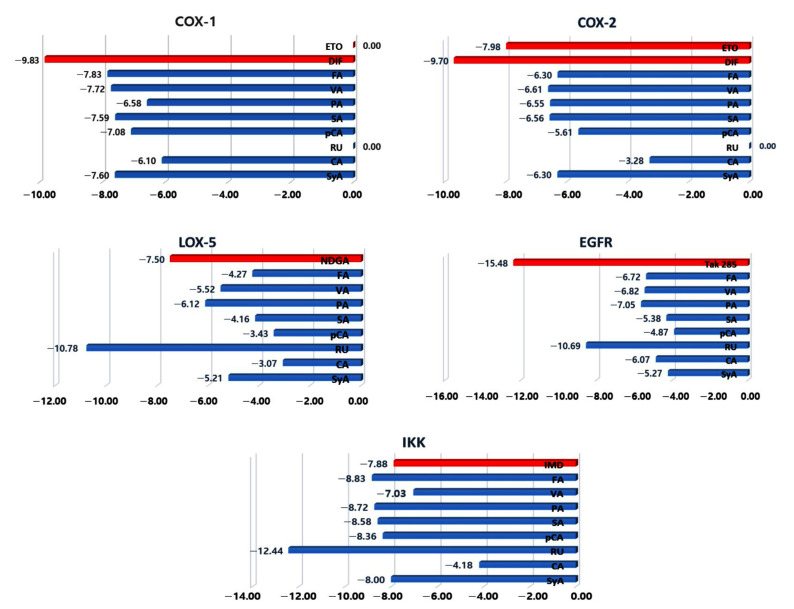
Docking score chart for selected compounds with each target. All the scores were transformed into positive values to create the chart. A value of 0.00 denotes no successful docking pose generated with the target active site. Red bars indicate the docking scores of the control compounds to the corresponding targets.

The molecular mechanism of inhibition of an enzyme often lies in the interaction between the inhibitor and the catalytic active site of the enzyme. Therefore, it is important to study the non-bond interactions of a target-ligand complex. In this study, we selected four major pathways for inflammation consisting of five key proteins (Figure 1). All the isolated compounds were docked to the five different proteins, namely COX-1, COX-2, LOX-5, EGFR, and IKK (Figure 4). [24]. Comprehensive data of the docking scores exhibit that PA and VA are the most active multi-target inhibitor with docking scores <−5.5 (Figure 4).

It is also perceivable that almost all the compounds mostly target the COX pathways as a primary means of anti-inflammatory activity. This observation is coherent to the in vivo writhing test. For COX-1, the highest docking score of −7.8 was for ferulic acid (FA), while the control compound diclofenac had −9.83 (Figure 4). As expected, etoricoxib (ETO) had no interaction with the enzyme. Both diclofenac (DIF) and ETO had docking scores of −9.70 and −7.98, respectively, while, among the tested compounds, VA had the highest docking score (−6.61) with COX-2 (Figure 4). LOX-5 was affected by RU, PA, and VA, while the standard inhibitor nordihydroguaiaretic acid (NDGA) [25] showed a score of −7.50 (Figure 4). EGFR showed robust docking scores with all except SyA and p-coumaric acid (pCA), while the control compound Tak285 (EGFR inhibitor) [26] exhibited a score of −15.48 (Figure 4). Only CA generated a poor docking score while others had <−7 with IKK. The control compound N-(3,5-Bis(trifluoromethyl)phenyl)-5-chloro-2-hydroxybenzamide (IMD) had a docking score of 7.88.

All compounds for each target, with docking scores ≤ −5.5, were selected for analyzing molecular interaction and molecular dynamics simulation. Non-bonding interaction analysis for all four targets revealed a good number of hydrogen bonds, hydrophobic interactions, electrostatic interaction, and some cases, halogen bonds with the fundamental molecules of the active sites (Table S2).

**Figure 5:**
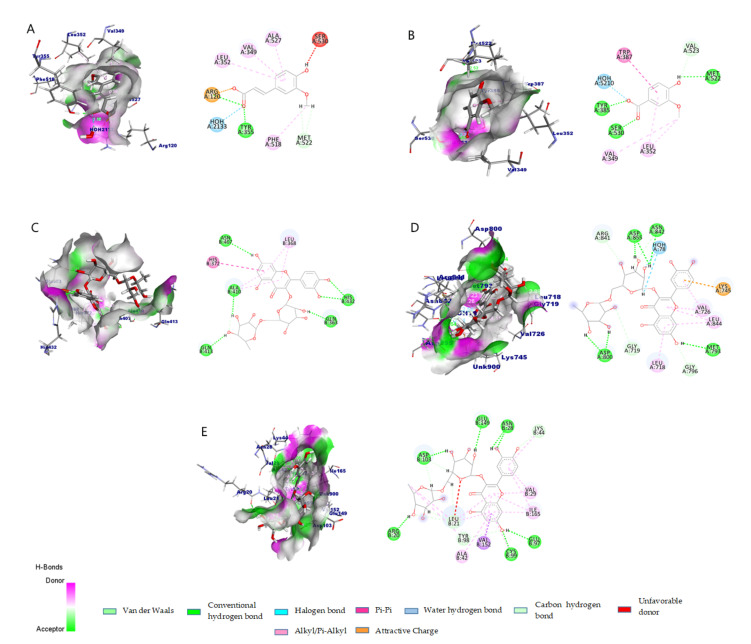
Graphical representation of non-bond interactions for top compounds with each target’s active site. (**A**–**E**) Represent the interactions for COX-1, COX-2, LOX-5, EGFR, and IKK respectively.

The COX-1 enzyme, with FA, VA, SyA, and the control, had interactions with crucial amino acids like Tyr 355, Ser 530, and Arg 120 (Figure 5A) for COX molecules [27]. Commonly occurred interactions for all selected and control compounds are with Arg 120, H2O 2133, Tyr 355, Leu 352, Val 349, and Ala 527. Interaction with Ser 530 is found only in the control DIF and FA. The lowest bond distance was 1.58 Å for Tyr 355 with FA.

On the contrary, the most common interaction for COX-2 was with Met 522. The minimum distance was 2.45 Å with SA. ETO, the control for COX-2 also showed interaction with Met 522, but the exception is that it was a hydrophobic interaction with a distance of 4.44 Å. In contrast, the others were hydrogen bonds (Table S2).

Rutin had the highest number (7 hydrogen and 5 hydrophobic bonds) of molecular interaction among the top three compounds with LOX-5 (Figure 5C). Interaction with Arg 596 was observed for all the compounds and the control. The minimum distance recorded was with VA (1.56 Å; Table S2).

Interactions for EGFR include hydrogen, and hydrophobic interactions were found for the compounds while only the control (Tak 285) exhibited the halogen bonds (Figure 5D). RU displayed the highest number of non-bond interactions, including electrostatic interaction, and it was 19 (Table S2). This result is consistent with the fact that this compound was found to be a potent kinase inhibitor of EGFR in the research of Seunghwan Choi and his team [24]. Among the top three compounds, other than RU, FA displayed the maximum number of interactions. Asp 793 was a commonplace for all compounds and the control. The lowest distance for this molecule was 1.99 Å with PA.

The isolated compounds, other than the CA, showed robust docking score with IKK. RU is the leading compound with both highest docking score and highest number (14 hydrogen bonds, 6 hydrophobic bonds) of non-bond interactions. The strongest hydrophobic interaction was observed between Val 152 and RU. FA, PA, SA, pCA, and VA displayed pi-sulfur bond with Met 96. However, no compound showed any electrostatic or halogen bonding similar to the control compound IMD. Leu 21, Val 29, Ala 42, Glu 97, Val 152, and Ile 165 are the common interacting residues for all compounds including the control.

In summary, molecular docking and interaction analyses strongly suggest that the extract of *MH* leaves can regulate inflammatory responses via EGFR and AA-mediated signaling pathways. Significantly, compounds PA and VA derived from the *MH* leaves are thought to function as potent inhibitors of multiple signaling mediators.

#### 2.2.5. Molecular Dynamics Simulation

**Figure 6:**
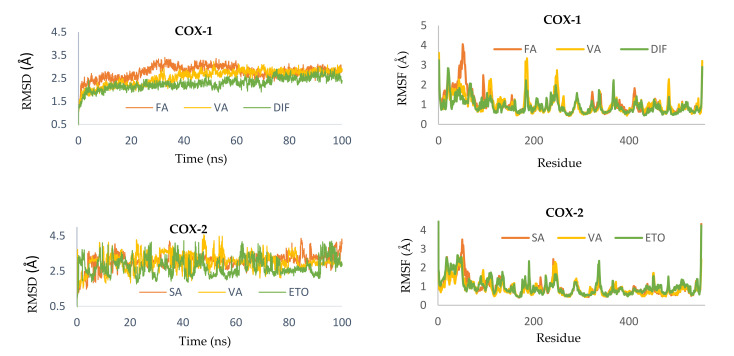

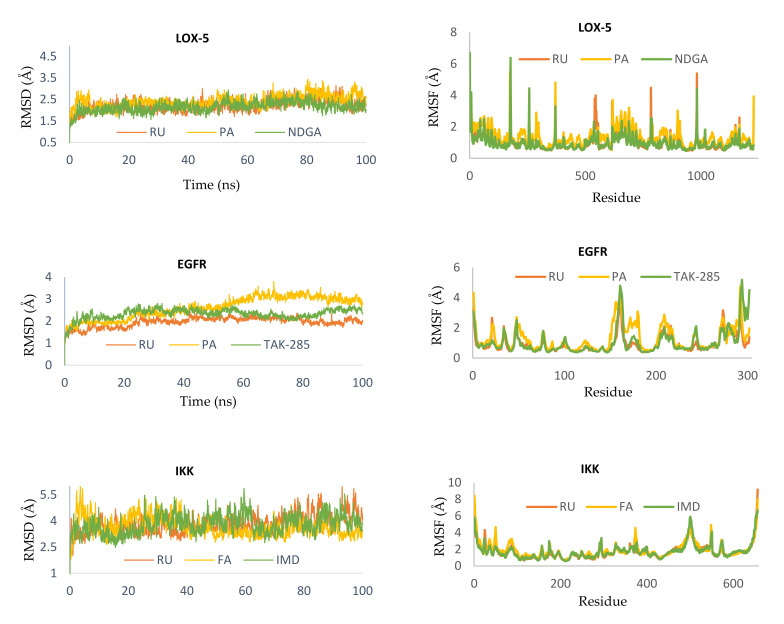
Superimposed RMSD graph of backbone and RMSF graph of residues for each target complexed with selected compounds and controls.

Determination of the binding stability and flexibility of ligand-protein complexes, Molecular dynamics (MD) simulations [28,29,30] were carried out for 100 nanoseconds (ns). For this study, we selected the top two candidates of each reaction with all five enzymes based on the docking scores and non-bond interaction analysis. The binding stability of the compounds and the residual flexibility of each protein-compound complex were analyzed by root mean squire deviation (RMSD) and root mean square fluctuation (RMSF), respectively (Figure 6). Ligand properties including radius of gyration (rGyr) and solvent accessible surface area (SASA) were compared with the control compound to identify the ligand extendedness and to assess the surface area exposed to the water molecule consecutively.

**Figure 7:**
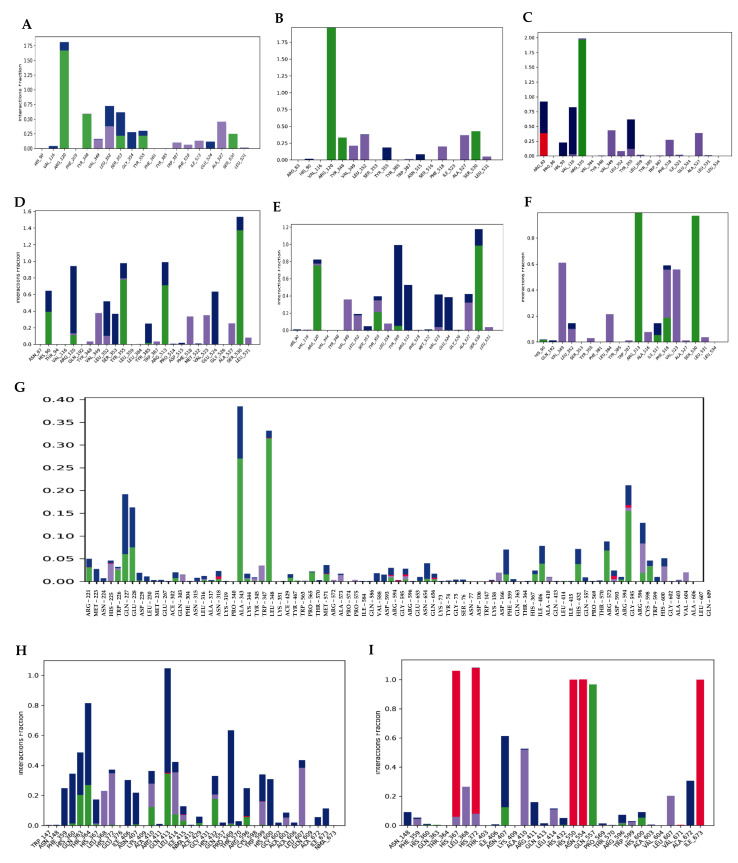

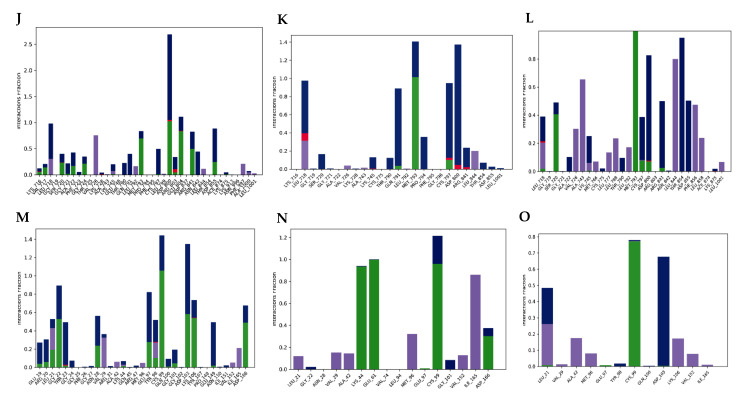
Interaction histogram. (**A**–**C**) COX-1 complexed with FA, VA, and DIF; (**D**–**F**) COX-2 complexed with SA, VA, and ETO; (**G**–**I**) LOX-5 complexed with PA, RU, and NDGA; (**J**–**L**) EGFR complexed with RU, PA, TAK-285; (**M**–**O**) IKK complexed with RU, FA, IMD.

**Figure 8:**
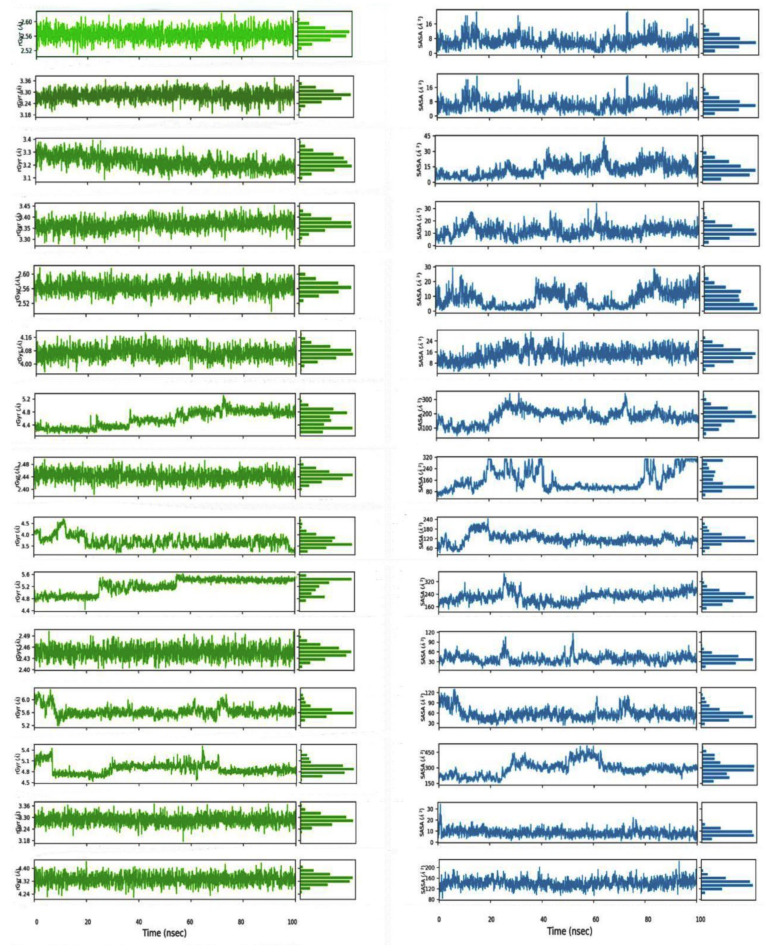
Ligand extendedness and accessible surface area by a water molecule through the 100 ns simulation production. Left column represent the radius of gyration (rGyr) data and right column exhibit the solvent accessible surface are (SASA) of the top two selected compounds and controls. The graphs are arranged in a sequence (top to bottom) for COX-1, COX-2, LOX-5, EGFR, and IKK.

The simulation for COX-1 showed that VA was most stable compared to FA and the control DIF. The system, complexed with VA, gets stable after ~38 ns and remains almost static till to the end. The control remains somewhat stable from 25 to 50 ns. The average RMSD for FA, VA, and DIF were 2.77, 2.55, and 2.28 Å, respectively. The spectrum of RMSD for VA was 2.55–3.22 Å. As for the RMSF, VA and DIF exhibit almost similar level of fluctuation. Arg120 is the central residue of the protein backbone to form hydrogen bonds with both compounds and the control (Figure 7). Although the rate for interaction with Arg120 was 100% for VA throughout the simulation, where for diclofenac, it was 99% (Figure S1). From the interaction timeline it is found that both FA and the control had multiple interactions with the Tyr355 (Figure S5). Both VA and FA exhibited steady rGyr values, whereas the control had three levels of fluctuations. The average value for VA was 2.56 Å and for FA, it was 3.27 Å. The early stage (0–27 ns) for DIF was stable for SASA. On the contrary, VA and FA had consistent graphs from ~20 to 60 ns (Figure 8).

No compound, including the control ETO, showed stability throughout the simulation. It seems to be that the system requires more time to be stable. The range of RMSD for SA, VA, and ETO were 0.90–4.36 Å, 1.28–4.56 Å, and 0.98–4.22 Å, respectively. Nevertheless, some insights can be derived from a close observation of the simulations. One of them is that SA outperformed control ETO by displaying a little bit of stability after ~40 ns up to ~57 ns. No such event was observed for the control. Ser530 is the common residue for all three compounds and the control to have a significant level of interaction. SA developed water bridge with Arg120, while there was no such interaction with either VA or ETO (Figure S2). The average spectrum of rGyr for VA, SA, and ETO are 2.55–2.59 Å, 3.34–3.39 Å, and 4.02–4.10 Å. This indicates that the control compound fluctuated most compared to the test compounds. SASA data implicate the compounds produced different level of stability for a 100 ns run. SA was stable from 65 ns to the end and VA was very briefly static from 22 to 38 ns. For the control it was 48 to 78 ns (Figure 8).

MD simulations of LOX-5 with RU, PA, and NDGA indicate that the system was somewhat stable with RU compared to others throughout the 100 ns run. The backbone residues of the protein showed almost similar level of RMSF for both compounds and the control. Only the control exhibits strong ionic bond with multiple amino acids whereas, highest number of hydrogen bonds were observed in PA (Figure 7). From the interaction timeline it can be said that PA failed to make significant level of interactions with the protein residues (Figure S6). NDGA showed water bridge with Asn407 for ligand-protein interaction while RU and PA had interactions with only His372 and Lys351 (Figure S3). Among the compounds and the control RU found to produce three stages of fluctuation for rGyr where the stage of 38 to 75 ns was the most irregular one. PA developed a consistent graph with the average fluctuation of 2.45 Å. The control, NDGA could produce a stable SASA reading from 58 to 86 ns with an average score of 130 Å. PA reached a stable state after 45 till 79 ns. RU showed such stability after 75 ns with an average fluctuation of 170 Å (Figure 8).

MD simulations of EGFR system showed that RU could produce stability since 56 ns to 79 ns. Both PA and the control failed to produce such static graph. The RMSF implies that all but RU cause a similar level of fluctuation of the backbone compared to the control Tak285. Met793 made both hydrogen bonds and water bridges with RU and PA while TAK-285 developed only hydrogen bond (Figure 7). Asp800 is the other amino acid which is involved in for both compounds and the control. There are several water bridges observed with Gln791, Asp800, and Thr854 (Figure S4). As for the rGyr, RU showed three phases of timeline. Among them the third phase, the most consistent and the longest, ranged from ~55 ns to till the end, and the average value was 5.4 Å. PA and the control produced much erratic graphs. From the perspective of SASA, both the compounds and the control showcased considerable stability. However, PA was most consistent with few fluctuations at 24 to 27 ns and 48 to 52 ns. The average score for RU, PA, and the control were 230 Å, 40 Å, and 60 Å respectively (Figure 8).

The IKK system was found more stable complexed with the RU while there were a few irregular peaks for FA and the control IMD. RU could produce a stable system from ~10 to ~32 ns. The range of RMSD value for RU 1.63 to 5.96 Å. Both compounds showed almost overlapped RMSF with the control. Cys99, Asp103, Lys106, and Asp166 are the commonplaces of interaction. Among them Glu97 and Asp103 involve water bridges (Figure S5). FA was found to show 99% ligand-protein contacts during the simulation. As for RU and control it was 59% and 70%, respectively, with Cys99. For both rGyr and SASA data, the control, IMD, and FA produced more static graphs throughout the simulation. Although RU produced some stability after 70 ns, the difference between average lowest value and average highest value is much lower compared to FA and IMD. The average rGyr and SASA for IMD, PA were 4.33 and 130 Å, 3.29 and 10 Å (Figure 8).

In conclusion, MD simulation of each selected target-compound complex suggests that the *MH* extract could regulate COX signaling pathway in a non-selective manner. In addition, some of the isolated compounds, such as RU is able to stabilize the LOX, EGFR, and IKK system with higher efficiency.

#### 2.2.6. ADMET Profile

Absorption, digestion, metabolism, and toxicity (ADMET) of the drug-like compounds from the extract were analyzed by the admetSAR tool. SyA showed the highest blood-brain barrier absorption with 58.6%, while SA, CA, and pCA also exhibit absorption ranged from 52.4% to 57.9% (Table 4). All the isolated compounds were predicted to be absorbed by the human intestine with a percentage of >80%. For Caco-2 permeability, all but CA and RU had predictions ranging 55.5% to 88.4%. There was a similar pattern for all compounds for metabolism. All of them predicted low cytochrome inhibitory promiscuity. No compound was found to be carcinogenic and toxic in the Ames test. The acute oral toxicity of SA, CA, pCA, RU, VA, and PA is class III, while FA and SyA are class IV and II, respectively.

Taken together, these findings indicate that it could be safe enough to use the *MH* extract as a treatment for inflammation because the isolated compounds are predicted to be non-carcinogenic with low oral acute toxicity and higher absorption to the intestine and brain tissues.

## 3. Discussion

The primary investigation, the writhing response experiment, was conducted to confirm the claim of analgesic effects of *MH* leaves extract. As there are no previously reported experimental data on this plant extract, we needed to confirm such activity by in vivo experiment and further build up the study using in silico methods. The acetic acid-induced writhing response is a painful procedure to evaluate peripherally acting analgesic activity in which the model represents pain sensation by triggering a localized inflammatory response. The response is thought to be mediated by acid-sensing ion channels and the prostaglandin pathways in peritoneal mast cells [31,32,33,34]. Although the concentration of extract required almost eight times higher than the regular analgesic drug to exhibit a similar level of writhing inhibition, it is confirmed that this extract is able to ameliorate injury-related pain or inflammation. The reason behind the higher concentration of extract may be due to the presence of different types of compounds in different concentrations.

Prediction of activity spectra for substances (PASS) of the selected compounds are similar to other derivatives of hydroxycinnamic acid, flavonoids, and hydroxy-benzoic acid [35,36,37]. This phenomenon suggests that these compounds have the potential to be used in analgesic or anti-inflammatory drug preparation. However, the druggability should be considered before prescribing as drugs. All but RU comply the RO5, yet RU is considered for further analysis for two reasons. First of all, its reported inhibition activity over EGFR; and finally, there are many exceptions found for RO5 [24,38].

Primary observation indicates that all the selected compounds are involved in the inhibition of both COX-1 and COX-2. Multiple key amino acids of both enzymes had non-bond interactions with the compounds. Especially, interaction with Tyr355, Tyr385, Ser530, and Arg120 indicates robust analgesic activity. Inhibition of the COX signaling pathway could explain the rapid writhing inhibition response by the extract. The cyclooxygenase-1 (COX-1) is reported to conduct the physiological actions while cyclooxygenase-2 (COX-2) is active in pro-inflammatory processes with a diverse class of enzymes downstream [39,40,41]. Therefore, overuse of the extract may lead to gastrointestinal injury. Although MD simulation data suggests that no selected compound could effectively stabilize the COX-2 system, regulation of EGFR with RU could reduce the side effects of NSAIDs by indirectly suppressing COX-2 expression. This phenomenon is already reported by Seunghwan Choi and his team [24]. In this work, the in silico study explored the molecular mechanism of such event. Inhibition of LOX-5 by RU can also reduce the concurrent side effect of NSAIDs to over activating of LOX-5 which leads to asthma [42,43,44]. Therefore, RU could be prescribed as supplementary along with NSAIDs for the patients who has history of asthma. Moreover, regulation of EGFR may influence the pro-inflammatory cytokines through inhibiting IKK-B mediated pathway. Although FA could not produce a stable RMSD graph, the strong interaction with some key amino acids like Cys99, Lys44, and Glu61 during the simulation makes it a viable compound. Further study on RU and FA could be conducted for IKK-B system. However, this study suggests that RU could be a strong candidate for direct influence over the IKK-B activity. In turn this property can be used to treat the conditions developed in diseases like COVID-19 infection due to cytokine storm [45].

There are some notable limitations in this study which should be mentioned. First of all, the in vivo experiment was conducted only for the analgesic activity. The other effects are predicted by utilizing computational tools only. No isolated compounds were used rather than the ethanolic crude extract. For the target-compound interaction, only the competitive inhibition was considered. However, allosteric inhibition may occur which is reserved for future investigation. Available computational tools limit our investigation to contemplate a single compound at a time, although, in biological environment multiple substrate may occupy the binding site at the same time. This study should be regarded as a primary prediction and subject to slight deviation when reproducing results in vitro and in vivo experiments. The results can be confidently taken as a guideline for further wet-lab testing and clinical trials.

## 4. Materials and Methods

This study includes both in vivo and computational experiments. Therefore, this section is divided into two distinct subsections. For in vivo analysis, living organisms and different chemicals were used, while for the computational approach, digital data from different reliable databases and multiple software and online tools were used.

### 4.1. In Vivo

#### 4.1.1. Collection and Identification of Specimen

Arrow Leafed Pondweed *Monochoria hastata* (family Pontederiaceae) was selected for this study due to its ethnomedicinal use in different parts of the world. Further, three important class of compounds namely, hydroxycinnamic acid derivatives, flavonoids, and hydroxy-benzoic acid derivatives are present in the plant extract; and these classes of compounds are well known for their anti-inflammatory properties [35,36,37]. The fresh mature leaves of were collected from Gazipur District of Bangladesh (Latitude: 24.095817 and Longitude: 90.412518; Latitude DMS: 24°5′44.94′’ N and Longitude DMS: 90°24′45.06′’ E). Identification and authentication of the plant sample has been done by the taxonomist and a voucher specimen (DACB-38364) was submitted to the national herbarium center Mirpur, Dhaka, Bangladesh.

#### 4.1.2. Chemicals, Drugs and Solvents

All the chemicals, including the solvents used, were of analytical grades. For analgesic test—acetic acid, diclofenac sodium, and etoricoxib INN 60 mg (was collected from Square Pharmaceuticals Ltd., Dhaka, Bangladesh). Other materials including, DMSO (Merck, Germany), Tween-80 as suspending agent (analytical grades.), and normal saline solution (Square Pharmaceutical Ltd., Bangladesh) were also used.

#### 4.1.3. Experimental Animals and Ethical Approval

The Swiss albino mice weighing (30.2 ± 2.9 g) of both sexes were used to conduct the research and procured from the animal research branch of International Centre for Diarrhoeal Disease Research, Bangladesh (icddr, b-International Centre for Diarrhoeal Disease and Research) Bangladesh. They kept under standard husbandry conditions (temperature Δ23 ± 2 °C RH = 55 ± 10 and 12 light and 12 h dark cycle). The animals were fed with commercial diet pellets and water ad libitum. Before the experimentation session, the animals were allowed to acclimatize to the atmosphere for seven days. Animals were kept fasting overnight but allowed free access to water. 

#### 4.1.4. Preparation of Extract

The *MH* leaves were collected and cleaned from undesirable plants or plant parts. The shade dried plants part ground into a coarse powder with the help of a suitable grinder. About 500 g of dried leaves powder was taken in a clean glass container, soaked in 4 L of ethanol, and associated with occasional shaking and stirring. The whole mixture then underwent a coarse filtration by a clean, white cotton and Whatman filter paper. The obtained filtrate evaporated and rendered the greenish-black color extract designated as crude extract of ethanol.

A total of 500 g of powdered leaves of *MH* was taken, and after evaporation, it yields 6.7 gm, so, percent yield was {(6.7/500) × 100%} = 1.34%.

#### 4.1.5. Analgesic/Anti-Nociceptive Activity by Acetic Acid Induced Writhing Method

Like conventional painkiller, the pain suppressive action of *MH* leaves is assayed by the acetic acid-induced writhing test 8. Intra-peritonea insertion of acetic acid (0.7% *v/v*) to the experimental animals creates a sensation of pain. Therefore, the animals squirm their body at regular intermission out of pain. This squirm or contraction of the body was termed “writhing.” As long as the animals feel pain, they continue to show writhing. Each writhing was counted and taken as an indication of pain sensation. Sometimes the animal did not accomplish a total writhing. This incomplete writhing was considered as half-writhing. Accordingly, two half-writhing were taken as one total writhing.

Any substance that has analgesic activity is supposed to lessen the number of the writhing of animals within a given time frame and for the control group. The writhing inhibition of positive control was taken as standard and compared with test samples and control. In the present study, Diclofenac and Etoricoxib were used as control drugs at a dose of 50 and 10 mg/kg bw, respectively (3% solution in sterile distilled water). Whereas *MH* extract was administrated at different concentrations orally. Twenty minutes after the administration of acetic acid, the writhing of each count and the percent of inhibition were calculated by comparing with the control group. The indication for analgesic activity was expressed as the decreasing number of writhing compared with the negative and positive control group.

#### 4.1.6. Experimental Design for In Vivo Analgesic Activity Assessment

The animals were divided into five groups (4 animals in each group), as Group 1: Negative control (Saline water treated); Group 2: Positive control ETO (Etoricoxib treated); Group 3: Positive control DIF (Diclofenac treated); Group 4: G-1 (*MH* 200 mg/kg bw); Group 5: G-2 (*MH* 400 mg/kg bw).

#### 4.1.7. Statistical Analysis

All experiments were reported as mean ± SEM (standard error of the mean). Statistical significance testing of the values obtained was performed by one-way analysis of variance (ANOVA). The group means were evaluated by Dunnet’s multiple comparisons for analgesic screening tests using the SPSS program (SPSS 20.0, USA). The data obtained were compared with the vehicle control group in all cases. Differences were considered statistically significant when *p* < 0.05 [46].

### 4.2. In Silico Analysis

#### 4.2.1. Receptor and Ligand Structure Acquisition

The X-ray crystallographic 3D structure of COX-2 (PDB code: 1PXX, 2.90 Å conjugated with Diclofenac), COX-1 (PDB code: 2AYL, 2.00 Å bound with Flurbiprofen), EGFR (PDB code: 3POZ, 1.50 Å complexed with Tak-285), LOX-5 (PDB code: 6N2W, 2.71 Å coupled with NDGA), and IKK (PDB code: 4KIK, 2.83 Å) were downloaded from online Protein Data Bank (https://www.rcsb.org/) (accessed on: 15 June 2021).

Different parts of *Monochoria hastata* (family Pontederiaceae), like leaf, stem, rhizome, root, and fruit, contain diverse chemical constituents. However, we considered the leaf ethanolic crude extract for testing analgesic activity by writhing test in mice model to ensure the traditional claim of pain reducing capabilities. We did not perform any LCMS and HPLC to identify the isolated compounds, we only focused and considered the isolated compounds from different fraction (ethanolic, methanolic, aqueous) data from previously reported in different scientific publications for computational assessment. Among various compounds we have selected specific compounds (like ferulic acid (FA), sinapic acid (SA), chlorogenic acid (CA), p- coumaric acid (pCA), rutin (RU), syringic acid (SyA), vanillic acid (VA), and protocatechuic acid (PA) on the basis of structure activity relationship (SAR) and druggability criteria) for in silico analysis [21,47]. All the structures of these isolated compounds and controls were downloaded from PubChem (https://pubchem.ncbi.nlm.nih.gov/) (accessed on: 15 June 2021) as SDF format.

#### 4.2.2. In Silico Prediction of Activity Spectra for Substances (PASS Prediction)

The biological potential based on the structure-activity relationship was evaluated using the online PASS program (http://www.pharmaexpert.ru/PASSonline/predict.php) (accessed on: 10 July 2021). To evaluate the pharmacological activities, PASS compares the desired structure with a training set composed of more than 205,000 compounds and can reveal more than 7200 types of biological effects [48]. This tool can predict the probability of being active (Pa) or being inactive (Pi) of a compound against a particular pharmacological action [49].

#### 4.2.3. Lipinski’s Rule of Five Parameters

The behavior of pharmacologically active compounds is affected mainly by the drug-likeness properties. These properties include molecular size, flexibility, hydrophobicity, number of hydrogen bond donors and hydrogen bond acceptors, and number of rotatable bonds. Compounds should comply with the drug-likeness for their medicinal use. Therefore, to check the drug-likeness, we assessed the isolated compounds using Lipinski’s five (RO5) rule. The assessment was conducted using an online tool—Molinspiration (http://www.molinspiration.com/cgi-bin/properties) (accessed on: 19 July 2021).

#### 4.2.4. Molecular Docking

- Protein Preparation

For molecular docking, each protein structure was prepared using the protein preparation wizard of the Maestro package. Fill in missing side chains using “prime” and “Cap termini” were selected for preprocessing. During preprocessing, any water molecule at a distance of 5 Å from the het group is removed. Several experiments showed that retaining water molecule in the minimum area of the binding site is important for forming hydrogen bonds [50]. A number of water molecules (1 to 7) were found to be retained in the binding site when visualized using visualizer tools. From the review and modification tab, all the symmetrical chains and other het groups were removed from the structure other than the ligand bound to the active site. The site surrounding the ligand was selected as the active site for molecular docking. Later, the structure was minimized using the OPLS3e force field [51].

- Ligand Preparation

Ligands were prepared directly from SDF format using the tool ligprep of the Maestro suite. The force field used was OPLS3e.

- Docking Simulation Validation

Re-docking of the native ligand into the inhibitor binding site of the receptor was done to validate the docking analysis, including calculations, reliability, and reproducibility.

- Molecular Docking

Molecular docking simulation was conducted using Glide (Maestro) [52]. The grid was generated with the “Receptor Grid Generation” tool by selecting the conjugated ligand to define the binding site. For docking precision, the extra precision (XP) option was selected [53].

- Docked Pose Analysis and Visualization

All the docked poses were visualized and analyzed using Discovery Studio (DS) 4.5 (Accelrys, San Diego, CA, USA).

#### 4.2.5. Molecular Dynamics (MD) Simulation

MD simulation was carried out by the Desmond of Maestro suite for each selected ligand–target complex and later compared with the control. The system for the simulation was built using the OPLS3e force field, and the system was neutralized by using 0.15 M NaCl. A recording interval of 50 ps was selected for the 50 ns MD simulation. After the simulation, a total number of 1000 frames had been recorded.

#### 4.2.6. ADMET Calculation

Determination of the safety of a compound can be carried out by ADMET profiling [54]. Computational methods have become a more practicable alternative to experimental ADMET profiling to be more cost-effective and less time-consuming in high-throughput drug discovery processes [55]. We predicted the ADMET properties of each isolated compound by admetSAR online database (http://lmmd.ecust.edu.cn/admetsar1/predict/) (accessed on: 6 August 2021).

## 5. Conclusions

In this study, we investigated the traditional perception of anti-inflammatory activity of *Monochoria hastata* leaves extract and the rational anti-inflammatory properties of the reported compounds. The in vivo result indicates the analgesic activity of the crude extract, while in silico analysis tried to focus on the molecular mechanism of action. Although no selective COX-2 inhibitor was found among the compounds, the activity of multiple compounds, for example, rutin, ferulic acid, and vanillic acid, can be further investigated by in vivo and in vitro analysis for multi-target drug design. From the computational study, it is clear that the extract primarily targets the COX pathway. Regulation of LOX-5, EGFR, and IKK by RU can be utilized to develop anti-inflammatory drugs where both NSAIDs-related side effects, like asthma, and cytokine storm in different types of chronic inflammation can be reduced.

## Figures and Tables

**Table 1:** The percentage of inhibition of writhing in acetic acid induced mice by analgesic activity of methanolic extract of *Monochoria hastata*.

Group	Number of Writhing ^1^				Mean	SD	SEM	Mean ± SEM	Inhibition (%)
	m1	m2	m3	m4					
Negative Control	32	31	29	33	31.25	1.71	0.85	31.25 ± 0.85	0
Positive control ETO (10 mg/kg bw)	6	8	6	7	6.75	0.95	0.48	6.75 ± 0.48	78.4
Positive control DIF (50 mg/kg bw)	11	8	10	9	9.5	1.29	0.65	9.5 ± 0.65	69.6
G-1 *(MH)* 200 mg/kg	19	23	20	21	20.75	1.71	0.85	20.75 ± 0.85	33.6
G-2 *(MH)* 400 mg/kg	13	14	14	17	14.5	1.73	0.87	14.50 ± 0.87	53.6

^1^ All values of writhing are mean ± SEM (number of mice in each group, *n* = 4).

**Table 2:** Reported compounds isolated from *MH* leaves extract.

Isolated Compound	PubChem ID	Canonical SMILES
Ferulic Acid (FA) ^1^	CID 445858	COC1=C(C=CC(=C1)C=CC(=O)O)O
Sinapic Acid (SA) ^1^	CID 637775	COC1=CC(=CC(=C1O)OC)C=CC(=O)O
Chlorogenic Acid (CA) ^1^	CID 1794427	C1C(C(C(CC1(C(=O)O)O)OC(=O)C=CC2=CC(=C(C=C2)O)O)O)O
p-Coumaric Acid (pCA) ^1^	CID 637542	C1=CC(=CC=C1C=CC(=O)O)O
Rutin (RU) ^2^	CID 5280805	CC1C(C(C(C(O1)OCC2C(C(C(C(O2)OC3=C(OC4=CC(=CC(=C4C3=O)O)O)C5=CC(=C(C=C5)O)O)O)O)O)O)O)O
Syringic Acid (SyA) ^2^	CID 10742	COC1=CC(=CC(=C1O)OC)C(=O)O
Vanillic Acid (VA) ^3^	CID 8468	COC1=C(C=CC(=C1)C(=O)O)O
Protocatechuic Acid (PA) ^3^	CID 72	C1=CC(=C(C=C1C(=O)O)O)O

^1^ Hydroxycinnamic acid derivatives, ^2^ flavonoids, ^3^ hydroxy-benzoic acid derivatives.

**Table 3:** Drug-like properties of isolated compounds.

Compound	TPSA (Å^2^)	MW ˂500	miLogP ˂5	HBD ˂5	HBA ˂10	*n*-ROTB ˂10	Lipinski’s Violation ˂1
FA	66.76	194.19	1.25	4	2	3	0
SA	76	224.21	1.26	2	5	4	0
CA	164.74	354.31	−0.45	6	9	5	1
pCA	57.53	164.16	1.43	2	3	2	0
RU	269.43	610.52	−1.06	10	16	6	3
SyA	76	198.17	1.2	2	5	3	0
VA	66.76	168.15	1.19	2	4	2	0
PA	77.75	154.12	0.88	3	4	1	0

**Table 4:** ADMET properties of the selected isolated compounds.

Parameters	FA	SA	CA	pCA	Rutin	SyA	VA	PA
BBB	−(53.1)	+(57.9)	+(56.6)	+(52.4)	−(85.4)	+(58.6)	−(51.5)	−(63.8)
HIA	+(96.1)	+(95.8)	+(74.3)	+(99.4)	+(80.4)	+(91.7)	+(92.3)	+(88.1)
Caco-2 permeability	+(71.8)	+(73.2)	−(80.1)	+(88.4)	−(91.7)	+(71.2)	+(70.6)	+(55.5)
CYP450 2C9 Substrate	No (74.6)	No (80.0)	No (79.0)	No (78.9)	No (76.4)	No (82.1)	No (77.2)	No (82.3)
CYP450 2D6 Substrate	No (89.2)	No (89.2)	No (89.8)	No (93.6)	No (89.6)	No (89.0)	No (89.1)	No (91.5)
CYP450 3A4 Substrate	No (62.9)	No (60.5)	No (54.9)	No (74.6)	No (53.7)	No (62.6)	No (64.8)	No (72.3)
CYP450 1A2 Inhibitor	No (75.1)	No (84.5)	No (90.5)	No (94.6)	No (86.7)	No (90.5)	No (88.6)	No (95.5)
CYP450 2C9 Inhibitor	No (57.9)	No (83.8)	No (90.7)	No (93.6)	No (90.7)	No (93.2)	No (81.3)	No (95.7)
CYP450 2D6 Inhibitor	No (95.9)	No (92.9)	No (93.9)	No (97.7)	No (95.5)	No (94.5)	No (97.0)	No (96.4)
CYP450 2C19 Inhibitor	No (62.8)	No (71.8)	No (90.7)	No (91.2)	No (90.3)	No (85.8)	No (82.5)	No (97.1)
CYP450 3A4 Inhibitor	No (92.4)	No (87.5)	No (87.4)	No (86.9)	No (92.5)	No (95.4)	No (97.1)	No (95.4)
CYP Inhibitory Promiscuity	Low (77.5)	Low (76.1)	Low (96.9)	Low (89.1)	Low (67.9)	Low (87.7)	Low (89.0)	Low (95.6)
AMES Toxicity	No (91.3)	No (90.2)	No (91.3)	No (95.2)	No (51.2)	No (93.4)	No (94.2)	No (93.3)
Carcinogens	No (90.8)	No (88.5)	No (93.4)	No (82.5)	No (96.1)	No (88.1)	No (90.5)	No (91.5)
Acute Oral Toxicity	IV (62.7)	III (45.0)	III (77.8)	III (49.0)	III (59.7)	II (47.7)	III (49.2)	III (50.6)
Carcinogenicity (Three-class)	No (59.0)	No (67.0)	No (61.3)	No (60.3)	No (67.4)	No (71.6)	No (62.9)	No (62.2)

BBB: Blood–brain barrier; HIA: Human intestinal absorption; CYP450: Cytochrome P450. The acute oral toxicity (according to US Environmental Protection Agency criteria) Class I: LD50 value is ≤50 mg/kg; Class II: LD50 value is >50 to 500 mg/kg; Class III: LD50 value is >500 to 5000 mg/kg.

## Data Availability

The data used to support the findings of this study are within the article and in Supplementary Materials.

## Supplementary Materials

The following are available online. Figure S1: Non-bond ligand-protein interaction for COX-1 with FA, VA, and DIF after 100 ns molecular dynamics simulation, Figure S2: Non-bond ligand-protein interaction for COX-2 with VA, PA, SA, and contorl ETO after 100 ns molecular dynamics simulation, Figure S3: Non-bond ligand-protein interaction for LOX-5 with RU, PA, and contorl NDGA after 100 ns molecular dynamics simulation, Figure S4: Non-bond ligand-protein interaction for EGFR with RU, PA, and contorl Tak-285 after 100 ns molecular dynamics simulation, Figure S5: Non-bond ligand-protein interaction for IKK with RU, FA, and contorl IMD after 100 ns molecular dynamics simulation, Figure S6: Per residue interaction timeline of the protein with the ligands in different time frame, Table S1: Pharmacological activity prediction for the isolated compounds, Table S2: Non-bond interaction of selected compounds with each enzyme active site.

## Sample Availability

Samples are not available.
