# Supplementary material for: A Report on Multi-Target Anti-Inflammatory Properties of Phytoconstituents from Monochoria hastata (Family: Pontederiaceae)
Source: Molecules. 2021 Dec 6;26(23):7397. doi: 10.3390/molecules26237397 (PMC8658818; doi:10.3390/molecules26237397)
Supplement: Supplementary file 1 [file molecules-26-07397-s001.zip › molecules-1441258-supplementary.pdf]

Supplementary Data

Figures

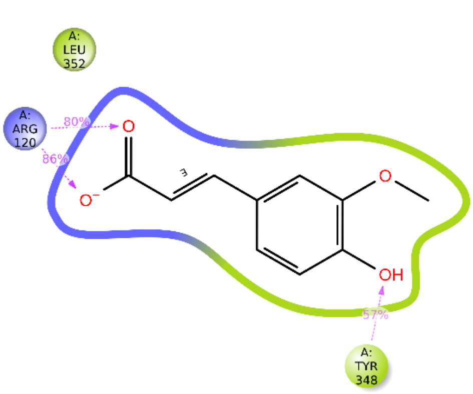

FA

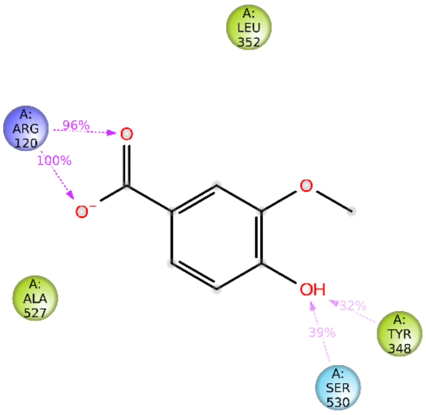

VA

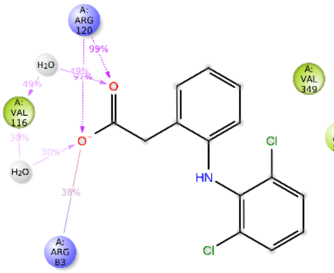

DIF

Figure S1. Non-bond ligand-protein interaction for COX-1 with FA, VA, and DIF after 100 ns mole cular dynamics simulation. Interaction that occurs more than 30% in the trajectory.

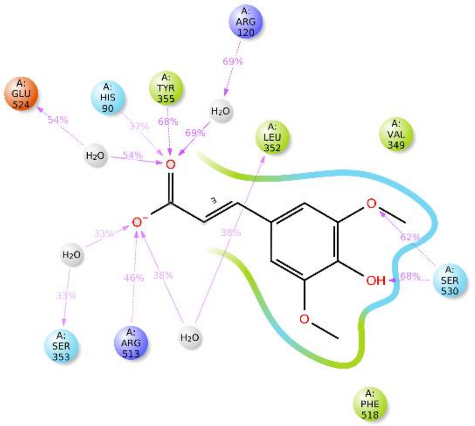

SA

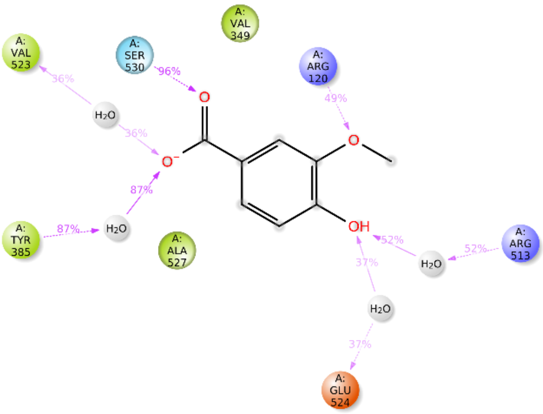

VA

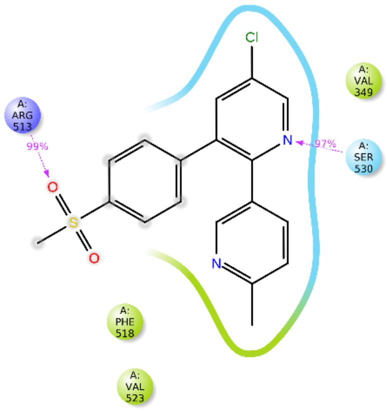

ETO

Figure S2. Non-bond ligand-protein interaction for COX-2 with VA, PA, SA, and contorl ETO after 100 ns molecular dynamics simulation. Interaction that occurs more than 30% in the trajectory.

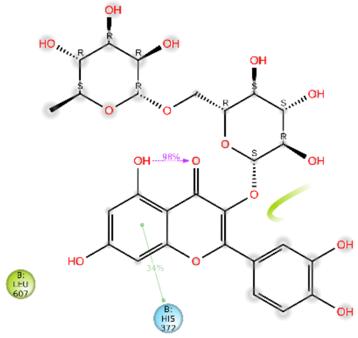

RU

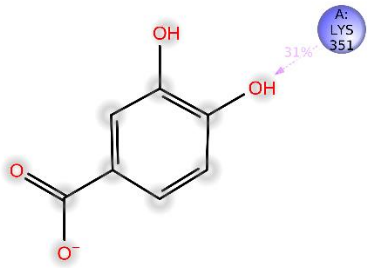

PA

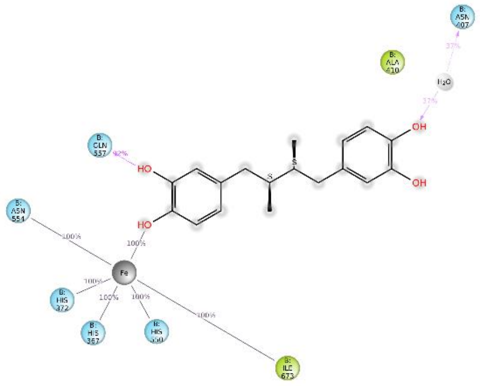

NDGA

Figure S3. Non-bond ligand-protein interaction for LOX-5 with RU, PA, and contorl NDGA after 1 00 ns molecular dynamics simulation. Interaction that occurs more than 30% in the trajectory.

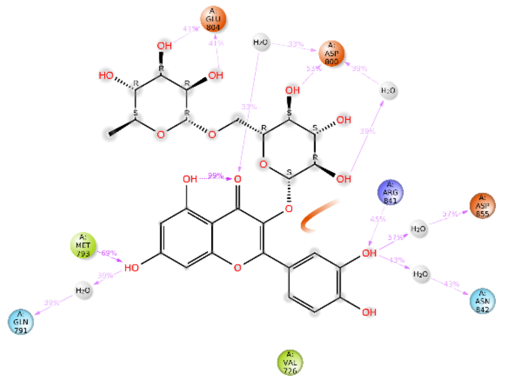

RU

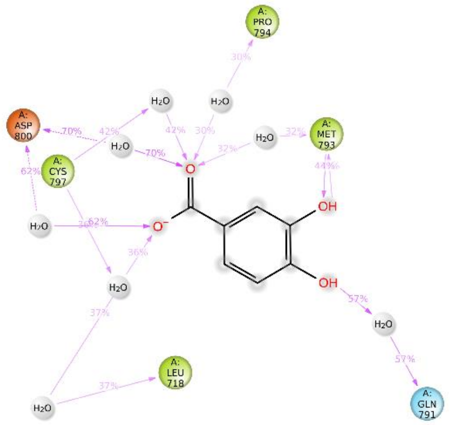

PA

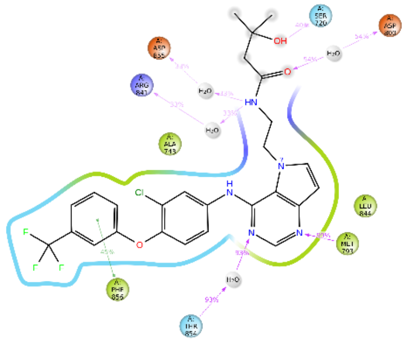

TAK-285

Figure S4. Non-bond ligand-protein interaction for EGFR with RU, PA, and contorl Tak-285 after 1 00 ns molecular dynamics simulation. Interaction that occurs more than 30% in the trajectory.

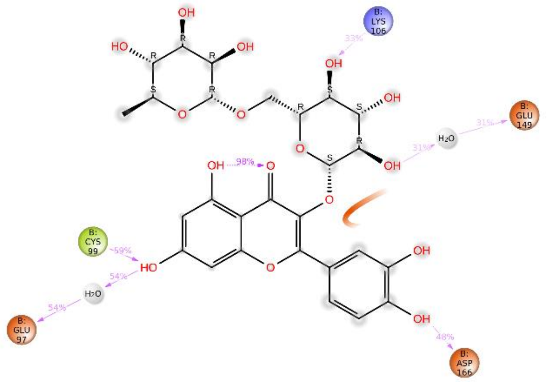

RU

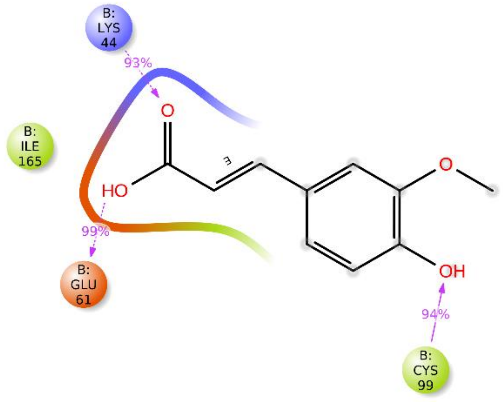

FA

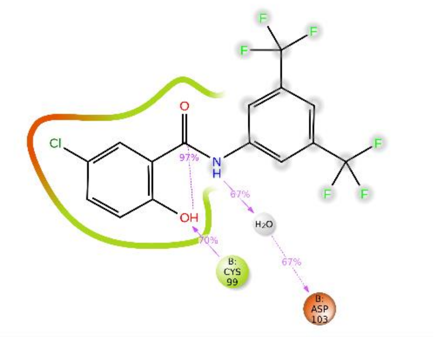

IMD

Figure S5. Non-bond ligand-protein interaction for IKK with RU, FA, and contorl IMD after 100 ns molecular dynamics simulation. Interaction that occurs more than 30% in the trajectory.

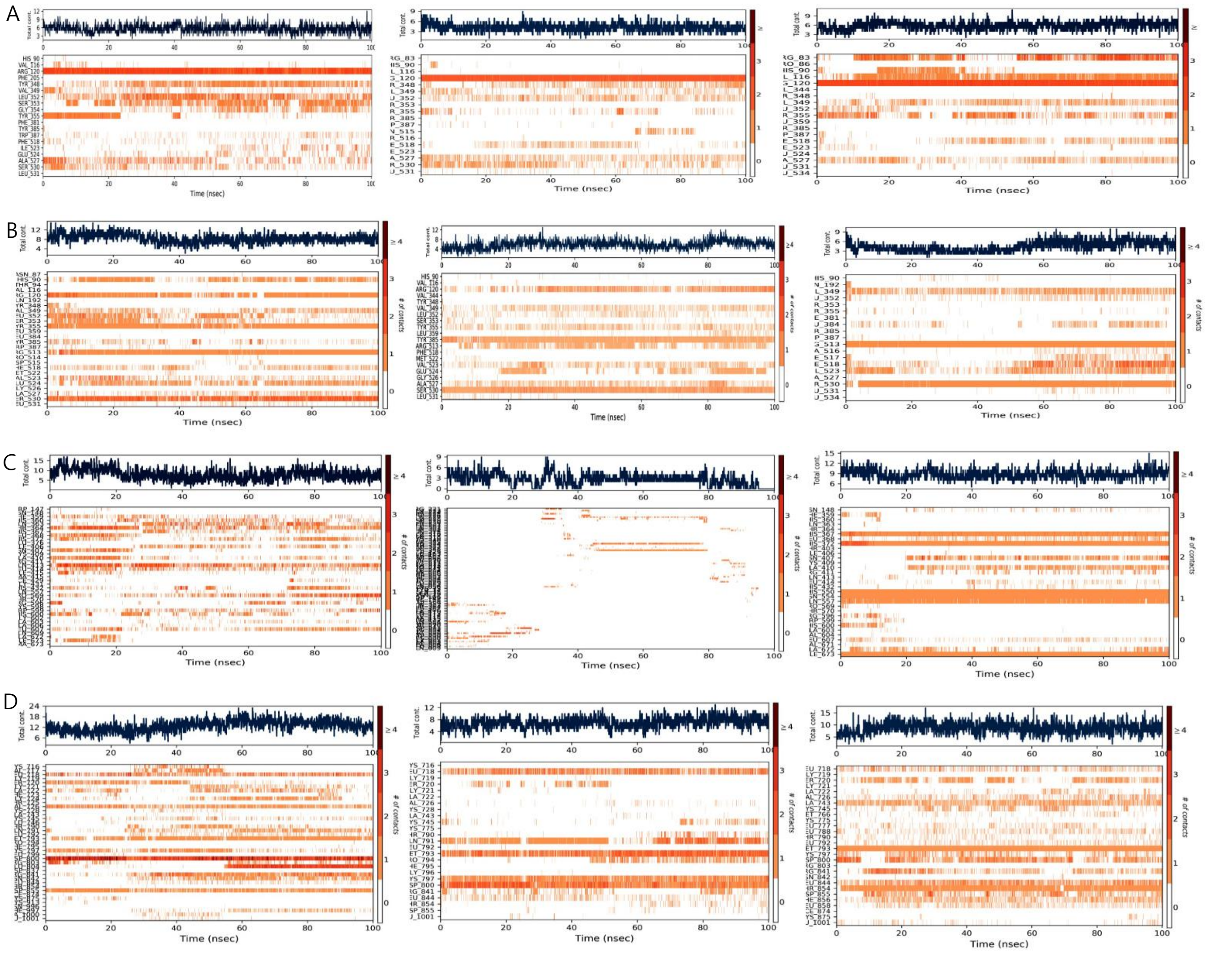

E

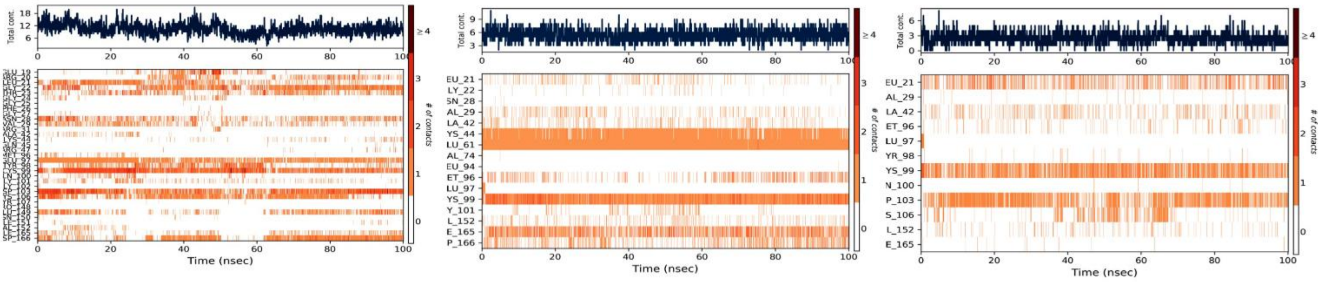

Figure S6. Per residue interaction timeline of the protein with the ligands in different time frame. Row A, B, C, D, and E represents the data of COX-1, COX-2, LOX-5, EGFR, and IKK respectively. A: (left to right) FA, VA, DIF; B: (left to right) SA, VA, ETO; C: (left to right) RU, PA, NDGA; D: (left to right) RU, PA, TAK-285; E: RU, FA, IMD.

Tables

Table S1. Pharmacological activity prediction for the isolated compounds. Pa> 0.7 was selected

Ferulic acid	Sinapic acid	Chlorogenic acid	p-Coumaric	Rutin	Syringic	Vanilic acid	Protocatechuic
Membrane integrity agonist	Feruloyl esterase inhibitor	Membrane integrity agonist	Feruloyl esterase inhibitor	Hemostatic	Chlordecone reductase inhibitor	Chlordecone reductase inhibitor	Chlordecone reductase inhibitor
JAK2 expression inhibitor	Membrane integrity agonist	Choleretic	Membrane integrity agonist	Membrane permeability inhibitor	Aspulvinone dimethylallyltransferase inhibitor	Aldehyde oxidase inhibitor	Dehydro-L-gulonate decarboxylase inhibitor
Mucomembranous protector	Antimutagenic	Membrane permeability inhibitor	Benzoate 4-monooxygenase inhibitor	Free radical scavenger	Ubiquinol-cytochrome-c reductase inhibitor	Feruloyl esterase inhibitor	Arylacetonitrilase inhibitor
Chlordecone reductase inhibitor	JAK2 expression inhibitor	Free radical scavenger	Mucomembranous protector	Cardioprotectant	4-Methoxybenzoate monooxygenase (O-demethylating) inhibitor	Aspulvinone dimethylallyltransferase inhibitor	Glutathione thiolesterase inhibitor
Aryl sulfotransferase inhibitor	Aspulvinone dimethylallyltransferase inhibitor	Lipid peroxidase inhibitor	Catechol oxidase inhibitor	Lipid peroxidase inhibitor	2-Hydroxyquinoline 8-monooxygenase inhibitor	2-Hydroxyquinoline 8-monooxygenase inhibitor	Alkane 1-monooxygenase inhibitor
Preneoplastic conditions treatment	Preneoplastic conditions treatment	Oxidoreductase inhibitor	Chlordecone reductase inhibitor	Membrane integrity agonist	Dehydro-L-gulonate decarboxylase inhibitor	Ubiquinol-cytochrome-c reductase inhibitor	Testosterone 17beta-dehydrogenase (NADP+) inhibitor
Antimutagenic	Mucomembranous protector	Anticarcinogenic	Benzoylformate decarboxylase inhibitor	CYP1A inducer	Testosterone 17beta-dehydrogenase (NADP+) inhibitor	Catechol 1,2-dioxygenase inhibitor	Threonine aldolase inhibitor
Aspulvinone dimethylallyltransferase inhibitor	Chlordecone reductase inhibitor	Chemopreventive	CYP2J substrate	Anticarcinogenic	Taurine dehydrogenase inhibitor	4-Methoxybenzoate monooxygenase (O-demethylating) inhibitor	Sugar-phosphatase inhibitor
Benzoate 4-monooxygenase inhibitor	Benzoate 4-monooxygenase inhibitor	HIF1A expression inhibitor	Pyruvate decarboxylase inhibitor	Vasoprotector	Aldehyde oxidase inhibitor	Dehydro-L-gulonate decarboxylase inhibitor	Arylsulfate sulfotransferase inhibitor
Mucositis treatment	Aryl sulfotransferase inhibitor	Phosphatase inhibitor	Antimutagenic	CYP1A1 inducer	Feruloyl esterase inhibitor	Shikimate 5-dehydrogenase inhibitor	Alkenylglycerophosphocholine hydrolase inhibitor
Linoleate diol synthase inhibitor	Diphosphomevalonate decarboxylase inhibitor	Antieczematic	Protocatechuate 3,4-dioxygenase inhibitor	Xanthine dehydrogenase inhibitor	Catechol 1,2-dioxygenase inhibitor	Arylacetonitrilase inhibitor	NADPH-cytochrome-c2 reductase inhibitor
MMP9 expression inhibitor	Ubiquinol-cytochrome-c reductase inhibitor	Antioxidant	Aspulvinone dimethylallyltransferase inhibitor	Hepatoprotectant	Sugar-phosphatase inhibitor	Preneoplastic conditions treatment	Glucan endo-1,6-beta-glucosidase inhibitor
Choleretic	4-Coumarate-CoA ligase inhibitor	Proliferative diseases treatment	3,4-Dihydroxyphenylacetate 2,3-dioxygenase inhibitor	Chemopreventive	NADPH-cytochrome-c2 reductase inhibitor	Taurine dehydrogenase inhibitor	2-Hydroxyquinoline 8-monooxygenase inhibitor
4-Coumarate-CoA ligase inhibitor	MMP9 expression inhibitor	Antineoplastic	Threonine aldolase inhibitor	UDP-glucuronosyltransferase substrate	Glutathione thiolesterase inhibitor	Glutathione thiolesterase inhibitor	Monodehydroascorbate reductase (NADH) inhibitor
Ubiquinol-cytochrome-c	Monophenol monooxygenase inhibitor	Feruloyl esterase inhibitor	GST A substrate	Monophenol monooxygenase inhibitor	Alkane 1-monooxygenase inhibitor	Vanillyl-alcohol oxidase inhibitor	Catechol 1,2-dioxygenase inhibitor

reductase inhibitor							
Vanillyl-alcohol oxidase inhibitor	Mucositis treatment	Quinate 5-dehydrogenase inhibitor	GABA aminotransferase inhibitor	Proliferative diseases treatment	Arylacetonitrilase inhibitor	Alkane 1-monooxygenase inhibitor	Ribulose-phosphate 3-epimerase inhibitor
Gluconate 2-dehydrogenase (acceptor) inhibitor	Gluconate 2-dehydrogenase (acceptor) inhibitor	Antihypoxic	Antiseborrheic	Histamine release stimulant	Methylenetetrahydrofolate reductase (NADPH) inhibitor	Testosterone 17beta-dehydrogenase (NADP+) inhibitor	Glutamyl endopeptidase II inhibitor
Protocatechuate 3,4-dioxygenase inhibitor	Beta-carotene 15,15'-monooxygenase inhibitor	Mucomembranous protector	JAK2 expression inhibitor	Morphine 6-dehydrogenase inhibitor	Ribulose-phosphate 3-epimerase inhibitor	Antiseptic	Cis-1,2-dihydro-1,2-dihydroxynaphthalene dehydrogenase inhibitor
Beta-carotene 15,15'-monooxygenase inhibitor	CYP2J substrate	4-Coumarate-CoA ligase inhibitor	Diphosphomevalonate decarboxylase inhibitor	Capillary fragility treatment	Superoxide dismutase inhibitor	Fibrinolytic	Methylenetetrahydrofolate reductase (NADPH) inhibitor
HIF1A expression inhibitor	Catechol oxidase inhibitor	G-protein-coupled receptor kinase inhibitor	4-Hydroxybenzoate 3-monooxygenase inhibitor	Anaphylatoxin receptor antagonist	Arylsulfate sulfotransferase inhibitor	Sugar-phosphatase inhibitor	UDP-N-acetylglucosamine 4-epimerase inhibitor
TNF expression inhibitor	Fumarate reductase (NADH) inhibitor	Beta-adrenergic receptor kinase inhibitor	NADPH peroxidase inhibitor	Antioxidant	Glucan endo-1,6-beta-glucosidase inhibitor	Vanillyl-alcohol oxidase inhibitor	Taurine dehydrogenase inhibitor
Membrane permeability inhibitor	Choleretic	UDP-glucuronosyltransferase substrate	Ubiquinol-cytochrome-c reductase inhibitor	CDP-glycerol glycerophosphotransferase inhibitor	UDP-N-acetylglucosamine 4-epimerase inhibitor	Alkenylglycerophosphocholine hydrolase inhibitor	Corticosteroid side-chain-isomerase inhibitor
Fumarate reductase (NADH) inhibitor	3-Demethylubiquinone-9 3-O-methyltransferase inhibitor	CDP-glycerol glycerophosphotransferase inhibitor	Dehydro-L-gulonate decarboxylase inhibitor	Antiprotozoal (Leishmania)	Fusarinine-C ornithinesterase inhibitor	Methylenetetrahydrofolate reductase (NADPH) inhibitor	Prolyl aminopeptidase inhibitor
2-Hydroxyquinoline 8-monooxygenase inhibitor	Carminative		NADPH-cytochrome-c2 reductase inhibitor	Antihypercholesterolemic	Threonine aldolase inhibitor	Threonine aldolase inhibitor	Aspartate-phenylpyruvate transaminase inhibitor
Carminative	TP53 expression enhancer		Phosphatidylcholine-retinol O-acyltransferase inhibitor	TP53 expression enhancer	Alkenylglycerophosphocholine hydrolase inhibitor	Arylsulfate sulfotransferase inhibitor	Pullulanase inhibitor
Catechol oxidase inhibitor	HMOX1 expression enhancer		Preneoplastic conditions treatment	Antidote	Alkenylglycerophosphocholine hydrolase inhibitor	Catechol 2,3-dioxygenase inhibitor	2-Nitropropane dioxygenase inhibitor
CYP2J substrate	Membrane permeability inhibitor		Mucositis treatment	UGT1A substrate	Fatty-acyl-CoA synthase inhibitor	Glucan endo-1,6-beta-glucosidase inhibitor	Aldehyde oxidase inhibitor
TP53 expression enhancer	GST A substrate		CYP4A11 substrate	Alpha glucosidase inhibitor	Shikimate 5-dehydrogenase inhibitor	Superoxide dismutase inhibitor	Fatty-acyl-CoA synthase inhibitor
Insulysin inhibitor	Linoleate diol synthase inhibitor		Arylsulfate sulfotransferase inhibitor	Aryl hydrocarbon receptor agonist	Spermidine dehydrogenase inhibitor	Ribulose-phosphate 3-epimerase inhibitor	Catechol 2,3-dioxygenase inhibitor
HMOX1 expression enhancer	Eye irritation, inactive		Fumarate reductase (NADH) inhibitor	2-Dehydropantoate 2-reductase inhibitor	Antiseptic	Membrane integrity agonist	Dextranase inhibitor
3-Demethylubiquinone-9 3-O-methyltransferase inhibitor	TNF expression inhibitor		Cis-1,2-dihydro-1,2-dihydroxynaphthalene dehydrogenase inhibitor	Beta-N-acetylhexosaminidase inhibitor	N-acylmannosamine kinase inhibitor	JAK2 expression inhibitor	Ubiquinol-cytochrome-c reductase inhibitor
Antiseptic	HIF1A expression inhibitor		Alkenylglycerophosphocholine hydrolase inhibitor	CYP3A4 inducer	Glutamyl endopeptidase II inhibitor	Linoleate diol synthase inhibitor	Gamma-guanidinobutyraldehyde dehydrogenase inhibitor
GST A substrate	2-Hydroxyquinoline 8-monooxygenase inhibitor		Glutathione thiolesterase inhibitor	NADPH oxidase inhibitor	Nitrate reductase (cytochrome) inhibitor	Fusarinine-C ornithinesterase inhibitor	Aspulvinone dimethylallyltransferase inhibitor
Fibrinolytic	NADPH peroxidase inhibitor		CYP2J2 substrate	Benzoate-CoA ligase inhibitor	Prolyl aminopeptidase inhibitor	UDP-N-acetylglucosamine 4-epimerase inhibitor	Benzoate 4-monooxygenase inhibitor
Sulfotransferase substrate	Insulysin inhibitor		Arylacetonitrilase inhibitor	Antineoplastic	5-O-(4-coumaroyl)-D-quinate 3'-monooxygenase inhibitor	Spermidine dehydrogenase inhibitor	Sulfite reductase inhibitor

Eye irritation, inactive	Antiseptic		Gluconate 2-dehydrogenase (acceptor) inhibitor	Caspase 3 stimulant	Pullulanase inhibitor	Beta-carotene 15,15'-monooxygenase inhibitor	Phloroglucinol reductase inhibitor
Monophenol monooxygenase inhibitor	Antihypoxic		Corticosteroid side-chain-isomerase inhibitor	HIF1A expression inhibitor	Aspartate-phenylpyruvate transaminase inhibitor	Fructose 5-dehydrogenase inhibitor	Feruloyl esterase inhibitor
Vasoprotector	Free radical scavenger		3-Phytase inhibitor	CYP2C9 inducer	Amine dehydrogenase inhibitor	Glutamyl endopeptidase II inhibitor	2-Dehydropantoate 2-reductase inhibitor
Caspase 3 stimulant	CYP2A6 substrate		APOA1 expression enhancer	CYP3A inducer	Fructose 5-dehydrogenase inhibitor	Amine dehydrogenase inhibitor	Bisphosphoglycerate phosphatase inhibitor
Steroid N-acetylglucosaminyltransferase inhibitor	Apoptosis agonist		Fatty-acyl-CoA synthase inhibitor	Kinase inhibitor	4-Nitrophenol 2-monooxygenase inhibitor	Cis-1,2-dihydro-1,2-dihydroxynaphthalene dehydrogenase inhibitor	Alcohol oxidase inhibitor
Antieczematic	Peroxidase inhibitor		Monophenol monooxygenase inhibitor	Lactase inhibitor	Arginine 2-monooxygenase inhibitor	Fatty-acyl-CoA synthase inhibitor	4-Methoxybenzoate monooxygenase (O-demethylating) inhibitor
Benzoylformate decarboxylase inhibitor	NADPH-cytochrome-c2 reductase inhibitor		Aspartate-phenylpyruvate transaminase inhibitor	CYP2H substrate	Monodehydroascorbate reductase (NADH) inhibitor	Nitrate reductase (cytochrome) inhibitor	Glucose oxidase inhibitor
CYP2E1 inducer	Pyruvate decarboxylase inhibitor		G-protein-coupled receptor kinase inhibitor	Chlordecone reductase inhibitor	Beta-carotene 15,15'-monooxygenase inhibitor	Prolyl aminopeptidase inhibitor	Arginine 2-monooxygenase inhibitor
Antihypoxic	Fibrinolytic		Beta-adrenergic receptor kinase inhibitor	P-benzoquinone reductase (NADPH) inhibitor	Antieczematic	5-O-(4-coumaroyl)-D-quinate 3'-monooxygenase inhibitor	Aminobutyraldehyde dehydrogenase inhibitor
Free radical scavenger	Antieczematic		MMP9 expression inhibitor	Antifungal	Aldehyde dehydrogenase (pyrroloquinoline-quinone) inhibitor	N-acylmannosamine kinase inhibitor	5-O-(4-coumaroyl)-D-quinate 3'-monooxygenase inhibitor
GABA aminotransferase inhibitor	Benzoylformate decarboxylase inhibitor		Testosterone 17beta-dehydrogenase (NADP+) inhibitor	Laxative	Peroxidase inhibitor	4-Nitrophenol 2-monooxygenase inhibitor	Sphinganine kinase inhibitor
Shikimate 5-dehydrogenase inhibitor	GABA aminotransferase inhibitor		2-Hydroxyquinoline 8-monooxygenase inhibitor	Mycothiol-S-conjugate amidase inhibitor	4-Hydroxyproline epimerase inhibitor	Corticosteroid side-chain-isomerase inhibitor	Aryl-alcohol dehydrogenase (NADP+) inhibitor
UDP-glucuronosyltransferase substrate	UDP-glucuronosyltransferase substrate		Linoleate diol synthase inhibitor	Trans-1,2-dihydrobenzene-1,2-diol dehydrogenase inhibitor	Anaphylatoxin receptor antagonist	Arginine 2-monooxygenase inhibitor	NADH kinase inhibitor
APOA1 expression enhancer	Vasoprotector		4-Coumarate-CoA ligase inhibitor	Beta glucuronidase inhibitor	Vanillyl-alcohol oxidase inhibitor	Pullulanase inhibitor	Sulfite oxidase inhibitor
Antiseborrheic	CYP2A substrate		Methylenetetrahydrofolate reductase (NADPH) inhibitor	Skin whitener	Corticosteroid side-chain-isomerase inhibitor	Aminomuconate-semialdehyde dehydrogenase inhibitor	Superoxide dismutase inhibitor
Pyruvate decarboxylase inhibitor	Saccharopepsin inhibitor		HIF1A expression inhibitor	HMOX1 expression enhancer	Cis-1,2-dihydro-1,2-dihydroxynaphthalene dehydrogenase inhibitor	Aspartate-phenylpyruvate transaminase inhibitor	Peptide alpha-N-acetyltransferase inhibitor
1-Acylglycerol-3-phosphate O-acyltransferase inhibitor	Acrocylindropepsin inhibitor		Beta-carotene 15,15'-monooxygenase inhibitor	Membrane integrity antagonist	Dextranase inhibitor	NADPH-ferrihemoprotein reductase inhibitor	Phosphatidylcholine-retinol O-acyltransferase inhibitor
Reductant	Chymosin inhibitor		Sulfite reductase inhibitor	NADPH-ferrihemoprotein reductase inhibitor	Bisphosphoglycerate phosphatase inhibitor	2-Nitropropane dioxygenase inhibitor	Glyoxylate oxidase inhibitor
Cytoprotectant	CYP2J2 substrate		Membrane permeability inhibitor	Glutathione-disulfide reductase inhibitor	3-Hydroxybenzoate 6-monooxygenase inhibitor	Monodehydroascorbate reductase (NADH) inhibitor	Antiseptic
Acrocylindropepsin inhibitor	Antiseborrheic		Phenylpyruvate decarboxylase inhibitor	Antiviral (Influenza)	Fibrinolytic	Peroxidase inhibitor	Magnesium-protoporphyrin IX monomethyl ester (oxidative) cyclase inhibitor
Chymosin inhibitor	Phobic disorders treatment		Pullulanase inhibitor	Radiosensitizer	Creatininase inhibitor	2-Dehydropantoate 2-reductase inhibitor	Membrane integrity agonist

MAP kinase stimulant			Prephenate dehydrogenase inhibitor	Apoptosis agonist	JAK2 expression inhibitor	Antimutagenic	3-Hydroxybenzoate 4-monooxygenase inhibitor
Saccharopepsin inhibitor			Peptide alpha-N-acetyltransferase inhibitor	Vasodilator	Sulfite reductase inhibitor	Aryl-alcohol dehydrogenase inhibitor	3-Hydroxybenzoate 6-monooxygenase inhibitor
Peroxidase inhibitor			Carminative	4-Coumarate-CoA ligase inhibitor	Phosphatidylcholine-retinol O-acyltransferase inhibitor	Aldehyde dehydrogenase (pyrroloquinoline-quinone) inhibitor	2,5-Dihydroxypyridine 5,6-dioxygenase inhibitor
3,4-Dihydroxyphenyl acetate 2,3-dioxygenase inhibitor			Sugar-phosphatase inhibitor	Histidine kinase inhibitor	NADH kinase inhibitor	Membrane permeability inhibitor	Procollagen-lysine 5-dioxygenase inhibitor
4-Hydroxybenzoate 3-monooxygenase inhibitor			CYP2C12 substrate	Xenobiotic-transporting ATPase inhibitor	Pro-opiomelanocortin converting enzyme inhibitor	Procollagen-lysine 5-dioxygenase inhibitor	Hyponitrite reductase inhibitor
Apoptosis agonist			Vanillyl-alcohol oxidase inhibitor	Radioprotector	Gamma-guanidinobutyraldehyde dehydrogenase inhibitor	Aminobutyraldehyde dehydrogenase inhibitor	NADPH-ferrihemoprotein reductase inhibitor
NADPH-cytochrome-c2 reductase inhibitor			Antihypoxic	Antiinflammatory	Aminobutyraldehyde dehydrogenase inhibitor	Dextranase inhibitor	Anthranilate 3-monooxygenase (deaminating) inhibitor
			Alkane 1-monooxygenase inhibitor	Cytostatic	Nicotinate dehydrogenase inhibitor	Trans-acenaphthene-1,2-diol dehydrogenase inhibitor	2-Hydroxymuconate-semialdehyde hydrolase inhibitor
			2-Dehydropantoate 2-reductase inhibitor	UGT1A7 substrate	Glucose oxidase inhibitor	4-Hydroxyproline epimerase inhibitor	Fusarinine-C ornithinesterase inhibitor
			Glucose oxidase inhibitor	Hepatic disorders treatment	Vanillyl-alcohol oxidase inhibitor	Sulfite reductase inhibitor	Creatininase inhibitor
			6-Pyruvoyltetrahydropterin synthase inhibitor	Nitric oxide antagonist	Antimutagenic	Sphinganine kinase inhibitor	N-benzyloxycarbonylglycine hydrolase inhibitor
			HMOX1 expression enhancer		Peptide alpha-N-acetyltransferase inhibitor	Gamma-guanidinobutyraldehyde dehydrogenase inhibitor	Dimethylargininase inhibitor
			Chorismate mutase inhibitor		Sphinganine kinase inhibitor	Bisphosphoglycerate phosphatase inhibitor	Protocatechuate 3,4-dioxygenase inhibitor
			2-Enoate reductase inhibitor		2-Hydroxymuconate-semialdehyde hydrolase inhibitor	Glucose oxidase inhibitor	Trans-acenaphthene-1,2-diol dehydrogenase inhibitor
			Protein-disulfide reductase (glutathione) inhibitor		Aryl-alcohol dehydrogenase inhibitor	Nicotinate dehydrogenase inhibitor	Taurocyamine kinase inhibitor
			NADH kinase inhibitor		Glutamate-5-semialdehyde dehydrogenase inhibitor	Mucositis treatment	Opheline kinase inhibitor
			Magnesium-protoporphyrin IX monomethyl ester (oxidative) cyclase inhibitor		Urethanase inhibitor	Glyoxylate oxidase inhibitor	Methylamine-glutamate N-methyltransferase inhibitor
			Vanillyl-alcohol oxidase inhibitor		tRNA-pseudouridine synthase I inhibitor	Peptide alpha-N-acetyltransferase inhibitor	Chenodeoxycholoyltaurine hydrolase inhibitor
			Arginine 2-monooxygenase inhibitor		2-Nitropropane dioxygenase inhibitor	Diphosphomevalonate decarboxylase inhibitor	Glutamine-phenylpyruvate transaminase inhibitor
			Sphinganine kinase inhibitor		Hyponitrite reductase inhibitor	Urethanase inhibitor	Electron-transferring-flavoprotein dehydrogenase inhibitor
			GABA C receptor agonist		Membrane integrity agonist	Aryl-alcohol dehydrogenase (NADP+) inhibitor	Rhamnulose-1-phosphate aldolase inhibitor
			Acrocylindropepsin inhibitor		Electron-transferring-flavoprotein dehydrogenase inhibitor	3-Hydroxybenzoate 6-monooxygenase inhibitor	Gentisate 1,2-dioxygenase inhibitor
			Chymosin inhibitor		Dimethylargininase inhibitor	Phosphatidylcholine-retinol O-	L-glucuronate reductase inhibitor

						acyltransferase inhibitor	
			Saccharopepsin inhibitor		Benzoate-CoA ligase inhibitor	4-Coumarate-CoA ligase inhibitor	Polyamine-transporting ATPase inhibitor
			Dextranase inhibitor		Methylamine-glutamate N-methyltransferase inhibitor	NADH kinase inhibitor	Nitrate reductase (cytochrome) inhibitor
			Choleretic		Glutamine-phenylpyruvate transaminase inhibitor	UGT1A6 substrate	2,6-Dihydroxypyridine 3-monooxygenase inhibitor
			Carboxypeptidase Taq inhibitor		Exoribonuclease II inhibitor	Nicotine dehydrogenase inhibitor	Crotonoyl-[acyl-carrier-protein] hydratase inhibitor
			TP53 expression enhancer		Phosphatidylserine decarboxylase inhibitor	Phosphatidylserine decarboxylase inhibitor	Long-chain-aldehyde dehydrogenase inhibitor
			Lipid metabolism regulator		Polyamine-transporting ATPase inhibitor	Pro-opiomelanocortin converting enzyme inhibitor	Glucan endo-1,3-beta-D-glucosidase inhibitor
			Phenylalanine dehydrogenase inhibitor		Gluconate 5-dehydrogenase inhibitor	Glutamate-5-semialdehyde dehydrogenase inhibitor	Phosphatidylserine decarboxylase inhibitor
			Reductant		Glyoxylate oxidase inhibitor	tRNA-pseudouridine synthase I inhibitor	Cyclohexyl-isocyanide hydratase inhibitor
			Glucan endo-1,6-beta-glucosidase inhibitor		Chenodeoxycholoyltaurine hydrolase inhibitor	Steroid N-acetylglucosaminyltransferase inhibitor	Gluconate 5-dehydrogenase inhibitor
			Insulysin inhibitor		Procollagen-lysine 5-dioxygenase inhibitor	Nitrite reductase [NAD(P)H] inhibitor	N-acetylneuraminate synthase inhibitor
			Ribulose-phosphate 3-epimerase inhibitor		L-glutamate oxidase inhibitor	Hyponitrite reductase inhibitor	Phenylpyruvate decarboxylase inhibitor
			Phobic disorders treatment		Lactaldehyde reductase inhibitor	2-Hydroxymuconate-semialdehyde hydrolase inhibitor	L-glutamate oxidase inhibitor
			Glycosylphosphatidylinositol phospholipase D inhibitor		Nicotine dehydrogenase inhibitor	Creatininase inhibitor	N-acetylneuraminate 7-O(or 9-O)-acetyltransferase inhibitor
			NADPH-ferrihemoprotein reductase inhibitor		Sarcosine oxidase inhibitor	Benzoate 4-monooxygenase inhibitor	Pterin deaminase inhibitor
			Glutamine-phenylpyruvate transaminase inhibitor		(R)-Pantolactone dehydrogenase (flavin) inhibitor	Long-chain-aldehyde dehydrogenase inhibitor	S-alkylcysteine lyase inhibitor
			Glutamyl endopeptidase II inhibitor		Crotonoyl-[acyl-carrier-protein] hydratase inhibitor	Phloroglucinol reductase inhibitor	Pyruvate decarboxylase inhibitor
			Methylamine-glutamate N-methyltransferase inhibitor		Membrane permeability inhibitor	Electron-transferring-flavoprotein dehydrogenase inhibitor	N-methylhydantoinase (ATP-hydrolysing) inhibitor
			Manganese peroxidase inhibitor		Opheline kinase inhibitor	Antiseborrheic	tRNA-pseudouridine synthase I inhibitor
			Dimethylargininase inhibitor		Taurocyamine kinase inhibitor	CYP2C12 substrate	Vanillyl-alcohol oxidase inhibitor
			Phosphatidylserine decarboxylase inhibitor		3-Demethylubiquinone-9 3-O-methyltransferase inhibitor	3-Demethylubiquinone-9 3-O-methyltransferase inhibitor	N-Acyl-D-aspartate deacylase inhibitor
			Fibrinolytic		Formaldehyde transketolase inhibitor	1,4-Lactonase inhibitor	Spermidine dehydrogenase inhibitor
			Phthalate 4,5-dioxygenase inhibitor		2-Dehydropantoate 2-reductase inhibitor	2,6-Dihydroxypyridine 3-monooxygenase inhibitor	Mucinaminylserine mucinaminidase inhibitor
			Allyl-alcohol dehydrogenase inhibitor		Sulfite oxidase inhibitor	N-acetylneuraminate 7-O(or 9-O)-acetyltransferase inhibitor	Ferredoxin-NAD+ reductase inhibitor

			Leukotriene-B4 20-monooxygenase inhibitor		Pterin deaminase inhibitor	Taurocyamine kinase inhibitor	Naphthalene 1,2-dioxygenase inhibitor	
			TNF expression inhibitor		ADP-thymidine kinase inhibitor	Opheline kinase inhibitor	N-acylmannosamine kinase inhibitor	
			Mucinaminylserine mucinaminidase inhibitor		Nitrite reductase [NAD(P)H] inhibitor	Dimethylargininase inhibitor	Urethanase inhibitor	
			Peroxidase inhibitor		NADPH-ferrihemoprotein reductase inhibitor	Glutamine-phenylpyruvate transaminase inhibitor	Aldehyde dehydrogenase (pyrroloquinoline-quinone) inhibitor	
			UDP-N-acetylglucosamine 4-epimerase inhibitor		1,4-Lactonase inhibitor	Methylamine-glutamate N-methyltransferase inhibitor	Histidinol-phosphatase inhibitor	
			Aminobutyraldehyde dehydrogenase inhibitor		N-benzyloxycarbonylglycine hydrolase inhibitor	Gluconate 2-dehydrogenase (acceptor) inhibitor	Tryptophanamidase inhibitor	
			1,4-Lactonase inhibitor		S-alkylcysteine lyase inhibitor	Polyamine-transporting ATPase inhibitor	Amine dehydrogenase inhibitor	
			Vasoprotector		Cyclohexyl-isocyanide hydratase inhibitor	Gluconate 5-dehydrogenase inhibitor	Glutamate-5-semialdehyde dehydrogenase inhibitor	
			Aryl sulfotransferase inhibitor		N-Acyl-D-aspartate deacylase inhibitor	Benzoate-CoA ligase inhibitor	Exoribonuclease II inhibitor	
			Antiseptic		Fructan beta-fructosidase inhibitor	L-glutamate oxidase inhibitor	Acetylesterase inhibitor	
			UGT1A7 substrate		Antiseborrheic	Insulysin inhibitor	Allyl-alcohol dehydrogenase inhibitor	
			Pectate lyase inhibitor		Long-chain-aldehyde dehydrogenase inhibitor	Chenodeoxycholoyltaurine hydrolase inhibitor	1,4-Lactonase inhibitor	
			Sulfotransferase substrate		Mucinaminylserine mucinaminidase inhibitor	Sulfite oxidase inhibitor	Phenol 2-monooxygenase inhibitor	
			Glucan 1,4-alpha-maltotetraohydrolase inhibitor		N-acetylneuraminate 7-O(or 9-O)-acetyltransferase inhibitor	Lactaldehyde reductase inhibitor	Poly(alpha-L-guluronate) lyase inhibitor	
			N-acetylneuraminate synthase inhibitor		UGT1A6 substrate	Aryl-acylamidase inhibitor	ADP-thymidine kinase inhibitor	
			Eye irritation, inactive		Naphthalene 1,2-dioxygenase inhibitor	Phenylpyruvate decarboxylase inhibitor	Aspartate-ammonia ligase inhibitor	
			UDP-glucuronosyltransferase substrate		Ferredoxin-NAD+ reductase inhibitor	Exoribonuclease II inhibitor	Glycerol dehydratase inhibitor	
			Exoribonuclease II inhibitor		Phenol O-methyltransferase inhibitor	(R)-Pantolactone dehydrogenase (flavin) inhibitor	Benzoate-CoA ligase inhibitor	
			Macrophage colony stimulating factor agonist		N-acetylneuraminate synthase inhibitor	Mucinaminylserine mucinaminidase inhibitor	Fructan beta-fructosidase inhibitor	
			S-alkylcysteine lyase inhibitor		Linoleate diol synthase inhibitor	2,5-Dihydroxypyridine 5,6-dioxygenase inhibitor	Fibrinolytic	
			Formaldehyde transketolase inhibitor		Phloroglucinol reductase inhibitor	Crotonoyl-[acyl-carrier-protein] hydratase inhibitor	4-Hydroxyproline epimerase inhibitor	
			Arylmalonate decarboxylase inhibitor		NADPH peroxidase inhibitor	N-methylhydantoinase (ATP-hydrolysing) inhibitor	Acylphosphatase inhibitor	
			CYP2A6 substrate		Glyoxylate reductase inhibitor	Gamma-butyrobetaine dioxygenase inhibitor	CYP2C12 substrate	
			Arylsulfatase inhibitor		Gluconate 2-dehydrogenase (acceptor) inhibitor	Cyclohexyl-isocyanide hydratase inhibitor	Lactaldehyde reductase inhibitor	
			4-Hydroxyphenylacetate 3-monooxygenase inhibitor		Lysase inhibitor	Gentisate 1,2-dioxygenase inhibitor	Carbon-monoxide dehydrogenase inhibitor	

			N-benzyloxycarbonyl glycine hydrolase inhibitor		N-methylhydantoinase (ATP-hydrolysing) inhibitor	Formaldehyde transketolase inhibitor	2-Oxoaldehyde dehydrogenase (NADP+) inhibitor
			Fusarinine-C ornithinesterase inhibitor		Aspartate-ammonia ligase inhibitor	Pterin deaminase inhibitor	Peroxidase inhibitor
			CYP4A substrate		2,6-Dihydroxypyridine 3-monooxygenase inhibitor	Phenol O-methyltransferase inhibitor	Formaldehyde transketolase inhibitor
			2,6-Dihydroxypyridine 3-monooxygenase inhibitor		Acetylesterase inhibitor	Lysase inhibitor	Arylalkyl acylamidase inhibitor
			N-formylmethionyl-peptidase inhibitor		Trans-acenaphthene-1,2-diol dehydrogenase inhibitor	3-Hydroxybenzoate 4-monooxygenase inhibitor	Phenol O-methyltransferase inhibitor
			Electron-transferring-flavoprotein dehydrogenase inhibitor		2-Oxoaldehyde dehydrogenase (NADP+) inhibitor	S-alkylcysteine lyase inhibitor	Fructose 5-dehydrogenase inhibitor
			Creatininase inhibitor		Mucositis treatment	N-Acyl-D-aspartate deacylase inhibitor	Preneoplastic conditions treatment
			Antihelmintic (Nematodes)		Carbon-monoxide dehydrogenase inhibitor	N-acetylneuraminate synthase inhibitor	Prostaglandin-A1 DELTA-isomerase inhibitor
			Glyoxylate oxidase inhibitor		CYP2C12 substrate	Mucomembranous protector	4-Nitrophenol 2-monooxygenase inhibitor
			Lysostaphin inhibitor		4-Coumarate-CoA ligase inhibitor	ADP-thymidine kinase inhibitor	Tpr proteinase (Porphyromonas gingivalis) inhibitor
			Acylcarnitine hydrolase inhibitor		3-Hydroxy-4-oxoquinoline 2,4-dioxygenase inhibitor	N-benzyloxycarbonylglycine hydrolase inhibitor	Uroporphyrinogen-III synthase inhibitor
			GST M substrate		Mannan endo-1,4-beta-mannosidase inhibitor	Naphthalene 1,2-dioxygenase inhibitor	Pro-opiomelanocortin converting enzyme inhibitor
			Monodehydroascorbate reductase (NADH) inhibitor		Tryptophanamidase inhibitor	Ferredoxin-NAD+ reductase inhibitor	Pectate lyase inhibitor
			Di-trans,poly-cis-decaprenylcistransferase inhibitor		Mannitol-1-phosphatase inhibitor	Fructan beta-fructosidase inhibitor	Xylan endo-1,3-beta-xylosidase inhibitor
			Aspartate-ammonia ligase inhibitor		Lysine 2,3-aminomutase inhibitor	Aryl sulfotransferase inhibitor	2-Oxoglutarate decarboxylase inhibitor
			Prostaglandin-E2 9-reductase inhibitor		Poly(alpha-L-guluronate) lyase inhibitor	Urease inhibitor	Astringent
			Alopecia treatment		Uroporphyrinogen-III synthase inhibitor	HIF1A expression inhibitor	Cholestanetriol 26-monooxygenase inhibitor
			5-O-(4-coumaroyl)-D-quinate 3'-monooxygenase inhibitor		Phenylpyruvate decarboxylase inhibitor	Acylphosphatase inhibitor	Guanidinoacetase inhibitor
			Venombin AB inhibitor		Salicylate 1-monooxygenase inhibitor	Hydroxylamine reductase (NADH) inhibitor	Centromere associated protein inhibitor
			All-trans-retinyl-palmitate hydrolase inhibitor		Allyl-alcohol dehydrogenase inhibitor	Glyoxylate reductase inhibitor	Glyoxylate reductase inhibitor
			ADP-thymidine kinase inhibitor		Glucan endo-1,3-beta-D-glucosidase inhibitor	Aspartate-ammonia ligase inhibitor	Mitochondrial processing peptidase inhibitor
			Antieczematic		Hydroxylamine reductase (NADH) inhibitor	2-Oxoaldehyde dehydrogenase (NADP+) inhibitor	Antiseborrheic
					Rhamnulose-1-phosphate aldolase inhibitor	Carbon-monoxide dehydrogenase inhibitor	Antieczematic
					Anthranilate-CoA ligase inhibitor	Mannan endo-1,4-beta-mannosidase inhibitor	Mannan endo-1,4-beta-mannosidase inhibitor

					Cholestanetriol 26-monooxygenase inhibitor	3-Hydroxy-4-oxoquinoline 2,4-dioxygenase inhibitor	Anthranilate-CoA ligase inhibitor
					3-Hydroxybenzoate 4-monooxygenase inhibitor	Glucan endo-1,3-beta-D-glucosidase inhibitor	Poly(beta-D-mannuronate) lyase inhibitor
					Aryl-alcohol dehydrogenase (NADP+) inhibitor	Rhamnulose-1-phosphate aldolase inhibitor	Limulus clotting factor B inhibitor
					Insulysin inhibitor	Prostaglandin-A1 DELTA-isomerase inhibitor	Nicotinate dehydrogenase inhibitor
					Limulus clotting factor B inhibitor	Salicylate 1-monooxygenase inhibitor	Transketolase inhibitor
					Benzoate 4-monooxygenase inhibitor	Alkenylglycerophosphoethanolamine hydrolase inhibitor	Aryldialkylphosphatase inhibitor
					Hydrogen dehydrogenase inhibitor	Tryptophanamidase inhibitor	N-carbamoyl-L-amino-acid hydrolase inhibitor
					L-glucuronate reductase inhibitor	Pyruvate decarboxylase inhibitor	Beta-carotene 15,15'-monooxygenase inhibitor
					Tpr proteinase (Porphyromonas gingivalis) inhibitor	Histidinol-phosphatase inhibitor	6-Pyruvoyltetrahydropterin synthase inhibitor
					Mitochondrial processing peptidase inhibitor	Mannitol-1-phosphatase inhibitor	Homoaconitate hydratase inhibitor
					3-Carboxyethylcatechol 2,3-dioxygenase inhibitor	Magnesium-protoporphyrin IX monomethyl ester (oxidative) cyclase inhibitor	
					Aminocarboxymuconate-semialdehyde decarboxylase inhibitor	MMP9 expression inhibitor	Biotin-CoA ligase inhibitor
					Steroid N-acetylglucosaminyltransferase inhibitor	Uroporphyrinogen-III synthase inhibitor	Triacetate-lactonase inhibitor
					Histidinol-phosphatase inhibitor	Orcinol 2-monooxygenase inhibitor	6-Carboxyhexanoate-CoA ligase inhibitor
					Fragilysin inhibitor	N-hydroxyarylamine O-acetyltransferase inhibitor	4-Chlorophenylacetate 3,4-dioxygenase inhibitor
					Polygalacturonase inhibitor	Pectate lyase inhibitor	Peptide-N4-(N-acetyl-beta-glucosaminyl)asparagine amidase inhibitor
					CYP2H substrate	Choleretic	L-amino-acid oxidase inhibitor
					Peptide-N4-(N-acetyl-beta-glucosaminyl)asparagine amidase inhibitor	Antiinflammatory, intestinal	NADPH peroxidase inhibitor
					Prostaglandin-A1 DELTA-isomerase inhibitor	Lysine 2,3-aminomutase inhibitor	Glycerol dehydrogenase (NADP+) inhibitor
					Pyruvate decarboxylase inhibitor	Limulus clotting factor B inhibitor	Catechol oxidase inhibitor
					Antiinfective	Monophenol monooxygenase inhibitor	Lysine 2,3-aminomutase inhibitor
					Xylan endo-1,3-beta-xylosidase inhibitor	Aryldialkylphosphatase inhibitor	3-Hydroxyphenylacetate 6-hydroxylase inhibitor
					6-Pyruvoyltetrahydropterin synthase inhibitor	Polygalacturonase inhibitor	Glutarate-semialdehyde dehydrogenase inhibitor
					D-xylulose reductase inhibitor	MAP kinase stimulant	DNA-(apurinic or apyrimidinic site) lyase inhibitor
					Glutarate-semialdehyde dehydrogenase inhibitor	UGT2B1 substrate	Linoleate diol synthase inhibitor
					4-Chlorophenylacetate 3,4-dioxygenase inhibitor	Acetylesterase inhibitor	Styrene-oxide isomerase inhibitor
					Astringent	Sarcosine oxidase inhibitor	3-Hydroxy-4-oxoquinoline 2,4-dioxygenase inhibitor

					Poly(beta-D-mannuronate) lyase inhibitor	CYP2H substrate	Arylsulfatase inhibitor
					Cyclohexanone monooxygenase inhibitor	H+-exporting ATPase inhibitor	Sarcosine oxidase inhibitor
					Gamma-butyrobetaine dioxygenase inhibitor	Hydrogen dehydrogenase inhibitor	Ornithine cyclodeaminase inhibitor
					Saccharopepsin inhibitor	APOA1 expression enhancer	Phenylacetate-CoA ligase inhibitor
					Acrocylindropepsin inhibitor	Tpr proteinase (Porphyromonas gingivalis) inhibitor	Glycerol 2-dehydrogenase (NADP+) inhibitor
					Chymosin inhibitor	Cytoprotectant	D-xylulose reductase inhibitor
					Triacetate-lactonase inhibitor	Anthranilate-CoA ligase inhibitor	Carnitinamidase inhibitor
					6-Carboxyhexanoate-CoA ligase inhibitor	L-glucuronate reductase inhibitor	Salicylate 1-monooxygenase inhibitor
					Homoaconitate hydratase inhibitor	Rubredoxin-NAD+ reductase inhibitor	Hydrogen dehydrogenase inhibitor
					Biotin-CoA ligase inhibitor	UGT1A9 substrate	Fragilysin inhibitor
					Aryldialkylphosphatase inhibitor	Poly(alpha-L-guluronate) lyase inhibitor	Mannitol-1-phosphatase inhibitor
					Magnesium-protoporphyrin IX monomethyl ester (oxidative) cyclase inhibitor	Cyclohexanone monooxygenase inhibitor	Creatinine deaminase inhibitor
					Alkenylglycerophosphoethanolamine hydrolase inhibitor	Antiinfective	2-Pyrocatechuate decarboxylase inhibitor
					Gluconolactonase inhibitor	Antipyretic	4-Hydroxyglutamate transaminase inhibitor
					Aryl-acylamidase inhibitor	Glycerol 2-dehydrogenase (NADP+) inhibitor	Alkenylglycerophosphoethanolamine hydrolase inhibitor
					H+-exporting ATPase inhibitor	Lipoprotein lipase inhibitor	Gluconolactonase inhibitor
					4-Hydroxyglutamate transaminase inhibitor	Chymosin inhibitor	3-Carboxyethylcatechol 2,3-dioxygenase inhibitor
					Arylalkyl acylamidase inhibitor	Saccharopepsin inhibitor	Aminocarboxymuconate-semialdehyde decarboxylase inhibitor
					Urease inhibitor	Acrocylindropepsin inhibitor	S-formylglutathione hydrolase inhibitor
					2,5-Dihydroxypyridine 5,6-dioxygenase inhibitor	NADPH peroxidase inhibitor	Antimutagenic
					Ornithine cyclodeaminase inhibitor	TP53 expression enhancer	3,4-Dihydroxy-9,10-secoandrosta-1,3,5(10)-triene-9,17-dione 4,5-dioxygenase inhibitor
					Diphosphomevalonate decarboxylase inhibitor	Prostaglandin-E2 9-reductase inhibitor	Opine dehydrogenase inhibitor
					Styrene-oxide isomerase inhibitor	Phobic disorders treatment	Leucolysin inhibitor
					Phospholipid-translocating ATPase inhibitor		Camphor 1,2-monooxygenase inhibitor
					S-formylglutathione hydrolase inhibitor		2-Enoate reductase inhibitor
					Diiodophenylpyruvate reductase inhibitor		Mucomembranous protector
					N-hydroxyarylamine O-acetyltransferase inhibitor		Nitrilase inhibitor
					(R)-6-hydroxynicotine oxidase inhibitor		Levanase inhibitor
					Kidney function stimulant		4-Phytase inhibitor
					Levanase inhibitor		Aryl-acylamidase inhibitor

					Carnitinamidase inhibitor		Diiodophenylpyruvate reductase inhibitor
					Histidine N-acetyltransferase inhibitor		D-alanine 2-hydroxymethyltransferase inhibitor
					Lipoprotein lipase inhibitor		Membrane permeability inhibitor
					Prostaglandin-E2 9-reductase inhibitor		CDP-4-dehydro-6-deoxyglucose reductase inhibitor
					TP53 expression enhancer		Mannan endo-1,6-alpha-mannosidase inhibitor
					Mucomembranous protector		3-Oxoadipate enol-lactonase inhibitor
					G-protein-coupled receptor kinase inhibitor		Thymidylate 5'-phosphatase inhibitor
					Beta-adrenergic receptor kinase inhibitor		2-Haloacid dehalogenase (configuration-inverting) inhibitor
					Phobic disorders treatment		Nicotine dehydrogenase inhibitor
							(R)-Pantolactone dehydrogenase (flavin) inhibitor
							Pyruvate dehydrogenase (cytochrome) inhibitor
							Trans-2-enoyl-CoA reductase (NAD+) inhibitor
							4,5-Dihydroxyphthalate decarboxylase inhibitor
							Glycerol-1-phosphatase inhibitor
							Trans-pentaprenyltranstransferase inhibitor
							Cyclomaltodextrinase inhibitor
							2-Dehydropantolactone reductase (A-specific) inhibitor
							Sulfite dehydrogenase inhibitor
							N-acetyl-gamma-glutamyl-phosphate reductase inhibitor
							4-Hydroxyphenylacetate 3-monooxygenase inhibitor
							Shikimate 5-dehydrogenase inhibitor
							P-benzoquinone reductase (NADPH) inhibitor
							N-hydroxy-2-acetamidofluorene reductase inhibitor
							Methylaspartate ammonia-lyase inhibitor
							Polygalacturonase inhibitor
							3,4-Dihydroxyphenylacetate 2,3-dioxygenase inhibitor
							Benzaldehyde dehydrogenase (NADP+) inhibitor

							Hydroxylamine reductase (NADH) inhibitor
							Nitrite reductase [NAD(P)H] inhibitor
							Mucositis treatment
							Quinate 5-dehydrogenase inhibitor
							Phosphoinositide 5-phosphatase inhibitor
							Pantoate 4-dehydrogenase inhibitor
							Ferredoxin-nitrite reductase inhibitor
							Phospholipid-translocating ATPase inhibitor
							D-amino-acid dehydrogenase inhibitor
							JAK2 expression inhibitor
							G-protein-coupled receptor kinase inhibitor
							Beta-adrenergic receptor kinase inhibitor
							Aminomuconate-semialdehyde dehydrogenase inhibitor
							Glycolate dehydrogenase inhibitor
							Phosphatidylglycerophosphatase inhibitor
							Quinoprotein glucose dehydrogenase inhibitor
							D-lactaldehyde dehydrogenase inhibitor
							Lysase inhibitor
							Inulinase inhibitor
							APOA1 expression enhancer
							Hippurate hydrolase inhibitor
							Histidine N-acetyltransferase inhibitor
							Glutamate decarboxylase inhibitor
							3-Hydroxybutyryl-CoA dehydrogenase inhibitor
							2,4-Diaminopentanoate dehydrogenase inhibitor

							Lysine 6-dehydrogenase inhibitor
							tRNA nucleotidyltransferase inhibitor
							Aspartyl aminopeptidase inhibitor
							Arylesterase inhibitor
							Prostaglandin-E2 9-reductase inhibitor
							Acetylornithine deacetylase inhibitor
							Benzoylformate decarboxylase inhibitor
							Glutaminase inhibitor
							N-Acyl-D-amino-acid deacylase inhibitor
							Nicotinamidase inhibitor
							Protocatechuate 4,5-dioxygenase inhibitor
							Lipoprotein lipase inhibitor
							Aryl-alcohol dehydrogenase inhibitor
							GABA C receptor agonist
							N-hydroxyarylamine O-acetyltransferase inhibitor
							Glycopeptide alpha-N-acetylgalactosaminidase inhibitor
							Phenylalanine 4-hydroxylase inhibitor
							Succinate-semialdehyde dehydrogenase [NAD(P)+] inhibitor
							(R)-6-hydroxynicotine oxidase inhibitor
							Laccase inhibitor
							Chitosanase inhibitor
							L-threonine 3-dehydrogenase inhibitor
							Penicillin amidase inhibitor
							2,3-Dihydroxyindole 2,3-dioxygenase inhibitor
							Peptide-tryptophan 2,3-dioxygenase inhibitor
							Bothrolysin inhibitor
							CYP2J substrate

							Beta-mannosidase inhibitor
							Protein-Npi-phosphohistidine-sugar phosphotransferase inhibitor
							Isopenicillin-N epimerase inhibitor
							Alcohol dehydrogenase (NADP+) inhibitor
							Carboxylate reductase inhibitor
							UGT1A6 substrate
							Phenylalanine(histidine) transaminase inhibitor
							Methylumbelliferyl-acetate deacetylase inhibitor
							Dopachrome isomerase inhibitor
							2,4-Dichlorophenol 6-monooxygenase inhibitor
							Beta-amylase inhibitor
							Phosphoglycerate mutase inhibitor
							Cyanoalanine nitrilase inhibitor
							Antiinfective
							CYP2J2 substrate
							Glucan endo-1,3-alpha-glucosidase inhibitor
							Lombricine kinase inhibitor
							H+-exporting ATPase inhibitor
							4-Coumarate-CoA ligase inhibitor
							Kidney function stimulant
							Dihydroxy-acid dehydratase inhibitor
							Leukotriene-B4 20-monooxygenase inhibitor
							Insulysin inhibitor
							Nicotinic alpha6beta3beta4alpha5 receptor antagonist
							Myosin ATPase inhibitor
							3-Aminobutyryl-CoA ammonia-lyase inhibitor
							Vomilenine glucosyltransferase inhibitor

							Indoleacetaldoxime dehydratase inhibitor
							Rubredoxin-NAD+ reductase inhibitor
							Glyceryl-ether monooxygenase inhibitor
							Pyruvate dehydrogenase (lipoamide) inhibitor
							Licheninase inhibitor
							Saccharopepsin inhibitor
							Chymosin inhibitor
							Acrocylindropepsin inhibitor
							Anaphylatoxin receptor antagonist
							Complement factor D inhibitor
							Chloramphenicol O-acetyltransferase inhibitor
							Cystathionine beta-synthase inhibitor
							Acetate kinase inhibitor
							Carnosine synthase inhibitor
							Prephenate dehydrogenase inhibitor
							Phenylacetaldehyde dehydrogenase inhibitor
							Orotate reductase (NADPH) inhibitor
							4-Hydroxybenzoate 3-monooxygenase inhibitor
							Alpha-N-acetylglucosaminidase inhibitor
							Biotinidase inhibitor
							Tannase inhibitor
							3-Cyanoalanine hydratase inhibitor
							L-iduronidase inhibitor
							3-Demethylubiquinone-9 3-O-methyltransferase inhibitor
							Urease inhibitor
							Gly-X carboxypeptidase inhibitor
							Leucine dehydrogenase inhibitor
							Phobic disorders treatment

							Protein-glutamate methylesterase inhibitor
							Thiosulfate dehydrogenase inhibitor
							HIF1A expression inhibitor
							Cyclamate sulfohydrolase inhibitor
							3-Phytase inhibitor
							X-methyl-His dipeptidase inhibitor
							N-(long-chain-acyl)ethanolamine deacylase inhibitor
							Carboxypeptidase Taq inhibitor
							Butyrate-CoA ligase inhibitor
							Plasmanylethanolamine desaturase inhibitor
							Steroid N-acetylglucosaminyltransferase inhibitor
							Porphobilinogen synthase inhibitor
							UGT2B1 substrate
							Glycosylphosphatidylinositol phospholipase D inhibitor
							Oxidoreductase inhibitor
							1-Alkylglycerophosphocholine O-acetyltransferase inhibitor
							O-aminophenol oxidase inhibitor
							Dimethylmaleate hydratase inhibitor
							Malate dehydrogenase (acceptor) inhibitor
							Gluconate 2-dehydrogenase (acceptor) inhibitor
							Ecdysone 20-monooxygenase inhibitor
							Manganese peroxidase inhibitor
							Cyclohexanone monooxygenase inhibitor
							4-Hydroxybenzoate 1-hydroxylase inhibitor
							DNA-3-methyladenine glycosylase I inhibitor
							Phthalate 4,5-dioxygenase inhibitor

							Aldehyde dehydrogenase (NADP+) inhibitor
							Cyclopentanone monooxygenase inhibitor
							Pitrilysin inhibitor
							Procollagen N-endopeptidase inhibitor
							4-Hydroxybenzoate decarboxylase inhibitor
							Glutathione dehydrogenase (ascorbate) inhibitor
							Coccolysin inhibitor
							Diphosphomevalonate decarboxylase inhibitor
							L-3-cyanoalanine synthase inhibitor
							Hydroxylamine reductase inhibitor
							Alcohol dehydrogenase [NAD(P)+] inhibitor
							Acyl-lysine deacylase inhibitor
							2,2-Dialkylglycine decarboxylase (pyruvate) inhibitor
							Succinate-semialdehyde dehydrogenase inhibitor
							Tyrosine-ester sulfotransferase inhibitor
							Gallate decarboxylase inhibitor
							Pyroglutamyl-peptidase II inhibitor
							HMOX1 expression enhancer
							L-ascorbate peroxidase inhibitor
							Oxalate oxidase inhibitor
							(S)-3-hydroxyacid ester dehydrogenase inhibitor
							Cytochrome-b5 reductase inhibitor
							Antiinflammatory, intestinal
							Glucan 1,4-alpha-maltotetraohydrolase inhibitor
							Cathepsin T inhibitor
							Glucan 1,6-alpha-glucosidase inhibitor
							Cholesterol antagonist

							Homogentisate 1,2-dioxygenase inhibitor
							Arylformamidase inhibitor
							Lipid metabolism regulator
							Arylmalonate decarboxylase inhibitor
							Erythropoiesis stimulant
							Aspartyltransferase inhibitor
							CYP2H substrate
							N-formylmethionyl-peptidase inhibitor
							Venombin AB inhibitor
							Acylcarnitine hydrolase inhibitor
							Nicotinic alpha2beta2 receptor antagonist

Table S2. Non-bond interaction of selected compounds with each enzyme active site

	Compound	Hydrogen Bond interaction			Hydrophobic Bond interaction			Electrostatic interaction			Halogen bond interaction/Pi-sulfur		
		No of bonds	Residues involved	Distance (Å)	No of bonds	Residues involved	Distance (Å)	No of bonds	Residues involved	Distance (Å)	No of bonds	Residues involved	Distance (Å)
COX-1	Ferulic acid (7.83)	6	H2O2133	2.18	4			1	Arg 120	3.59			
			Arg 120	1.69		Phe 518	4.77						
			Arg 120	2.04		Val 349	4.24						
			Tyr 355	1.58		Leu 352	5.20						
			Met 522	2.62		Ala 527	3.97						
			Met 522	2.35									
	Vanilic acid (7.72)	5	Arg 120	1.72	6	Leu 352	3.87						
			H2O2133	1.91		Ile 523	4.38						
			Arg 120	1.68		Phe 518	4.64						
			Tyr 355	1.60		Val 349	5.10						
			Leu 352	2.95		Ile 523	5.22						
						Ala 527	3.66						
	Syringic acid (7.60)	5			9	Ala 527	4.11	1	Arg 120	3.94			
						Leu 352	4.12						
			H2O2133	1.92		Ile 523	4.14						
			Arg 120	2.02		Val 349	3.82						
			Arg 120	1.86		Leu 531	3.71						
			Tyr 355	1.85		Phe 518	4.61						

			Leu 352	2.94		Val 349	5.04						
						Ile 523	5.21						
						Ala 527	3.68						
	Diclofenac (9.82)	5	Arg 120	1.98	6	Leu 352	5.37	1	Arg 120	3.52	1	Met 522	2.55
			H2O2133	2.02		Ile 523	4.00						
			Arg 120	1.54		Ala 527	4.67						
			Tyr 355	1.85		Val 349	4.96						
			Ser 530	2.29		Leu 352	4.91						
						Ala 527	5.19						
COX-2	Vanilic acid (6.61)	5	H2O 5210	2.28	4	Trp 387	5.35						
			Tyr 385	1.70		Val 349	4.78						
			Ser 530	1.82		Leu 352	3.97						
			Met 522	2.53		Leu 352	5.49						
			Val 523	2.56									
	Sinapic acid (6.56)	5			8	Gly 526, Ala 527	4.40	1	Arg 120	3.24			
			Tyr 355	2.06		Val 349	4.41						
			Ser 530	1.75		Leu 384	5.33						
			Ser 530	2.85		Met 522	4.66						
			Met 522	2.54		Tyr 348	4.72						
			Met 522	2.45		Val 349	4.95						
						Leu 352	5.45						
						Ala 527	4.54						
	Protocatechuic acid (6.55)	5	H2O 5210	2.29	1	Trp 387	5.37						
			Tyr 385	1.67									
			Ser 530	1.81									
			Met 522	2.52									
			Val 523	2.54									
	Etoricoxib (7.98)	3			13	Ser 353	2.00						
						Val 116	4.53						
						Leu 359	4.55						
						Leu 384	5.32						
						Met 522	4.44						
			Phe 518	2.86		Tyr 355	4.53						
			His 90	2.78		Trp 387	4.71						

			Ala 527	3.00		Phe 518	4.65				
						Val 349	4.64				
						Ala 527	3.79				
						val 523	4.67				
						Ala 527	4.40				
						val 523	3.48				
LOX-5	Rutin (10.78)	7	His 432	2.77	5						
			His 432	2.40		His 372	5.97				
			Gln 363	1.74		Leu 368	4.22				
			Ala 410	2.33		Ala 410	5.01				
			Gln 413	2.06		Leu 368	4.13				
			Asn 407	2.88		Ala 410	3.40				
			Ala 410	2.71							
	Syringic acid (5.21)	4	Arg 596	1.78	4	Ala 603	3.71				
			His 432	2.02		Val 604	4.86				
			Gln 363	2.07		Trp 599	4.99				
			His 600	2.72		His 600	4.47				
	Protocatechuic acid (6.12)	5	Arg 596	1.79	2						
			Arg 596	1.56		Phe 359	5.14				
			Gln 363	2.26		Ala 603	4.99				
			Gln 363	1.68							
			Gln 557	2.71							
	Vanilic acid (5.52)	3	Arg 596	1.56	2	Phe 359	5.36				
			Arg 596	1.76		Ala 603	5.09				
			Gln 363	1.90							
	NDGA (7.50)	5	His 372	2.86	6	Phe 359	5.16				
			Arg 596	2.01		Trp 599	5.32				
			Arg 596	2.13		Leu 607	5.40				
			Ile 406	2.45		Trp 599	5.48				
			His 600	2.06		Ala 410	4.41				
						Ala 603	5.13				
EGFR	Rutin (10.69)	12	H2O 78	2.44	6			1	Lys 745	4.91	
			H2O 78	2.58							
			Asp 855	2.09							
			Asn 842	2.64		Val 726	4.15				

			Asp 800	1.93		Leu 844	5.26						
			Asp 800	1.64		Leu 718	4.26						
			Met 793	2.10		Leu 844	5.23						
			Gly 719	2.41		Val 726	4.93						
			Gly 796	2.88		Lys 745	4.24						
			Arg 841	2.68									
			Asn 842	2.77									
			Lys 745	2.64									
	Protocatechuic acid (7.05)	4	H2O 14	2.13	3	Leu 718	4.86						
			Met 793	1.99		Ala 743	4.28						
			Gln 791	2.91		Leu 844	4.56						
			Leu 792	2.51									
	Vanilic acid (6.82)	4	H2O 14	2.02	5	Ala 743	3.93						
			Met 793	2.00		Val 726	4.91						
			Gln 791	2.69		Leu 718	5.05						
			Leu 792	2.62		Ala 743	4.21						
						Leu 844	4.42						
	Ferulic acid (6.72)	5	H2O 14	1.99	5	Ala 743	3.94						
			Met 793	2.00		Val 726	4.95						
			Gln 791	2.65		Leu 718	5.07						
			Gly 719	2.55		Ala 743	4.17						
			Leu 792	2.62		Leu 844	4.42						
	Tak 285 (15.48)	8			17	Phe 856	5.36				7		
						Lys 745	4.50						
						Leu 788	4.50						
						Cys 775	4.91						
						Phe 856	4.69						
			H2O 14	1.96		Phe 997	4.84					Thr 790	2.93
			H2O 78	2.51		Phe 997	5.22					Leu 788	2.96
			Thr 790	2.93		Leu 718	4.47					Met 766	3.26
			Met 793	2.07		Ala 743	4.41					Cys 775	2.85
			Cys 797	2.00		Leu 844	4.85					Cys 775	2.99
			Asp 800	1.73		Ala 743	3.35					Arg 776	3.38
			Thr 790	3.05		Leu 844	4.48					Arg 776	3.69
			Gln 791	2.20		Val 726	5.22						

						Lys 745	3.98				
						Met 766	4.54				
						Leu 777	5.37				
						Leu 788	5.46				
IKK	Rutin (12.44)	14	Asn 28	2.05	6	Val 152	2.75				
			Cys 99	2.12		Leu 21	4.95				
			Asp 103	1.96		Tyr 98	5.49				
			Glu 149	2.12		Leu 21	4.65				
			Arg 20	3.06		Val 29	4.87				
			Glu 97	2.22		Val 152	5.11				
			Asn 28	3.05		Ile 165	4.89				
			Leu 21	2.71		Leu 21	4.50				
			Lys 44	2.73		Ala 42	4.72				
			Tyr 98	2.55		Cys 99	5.13				
			Asp 103	2.11		Ile 165	5.40				
			Asp 103	2.33		Val 29	4.10				
			Leu 21	2.63		Lys 44	5.40				
			Leu 21	2.57		Ile 165	5.25				
	Ferulic acid (8.83)	2			7	Leu 21	4.16		1	Met 96	4.96
						Val 152	4.54				
			Glu 97	2.05		Val 29	5.47				
			Asp 166	2.49		Ala 42	3.90				
						Val 74	5.35				
						Val 152	4.89				
						Ile 165	4.49				
	Protocatechuic acid (8.72)	3			5	Leu 21	5.19		1	Met 96	5.49
			Cys 99	2.03		Val 29	5.43				
			Glu 97	1.96		Ala 42	4.05				
			Cys 99	2.00		Val 152	4.71				
						Ile 165	4.61				
	Sinapic acid (8.58)	3			5	Val 29	2.76		1	Met 96	5.12
			Cys 99	2.11		Lys 44	4.03				
			Glu 97	1.81		Leu 94	5.26				
			Tyr 98	2.72		Met 96	4.66				
						Ile 165	3.96				

	pCoumaric acid (8.36)	2			5	Ala 42	3.84				1	Met 96	4.98
			Glu 97	2.08		Val 74	5.34						
			Asp 166	2.54		Cys 99	5.48						
						Val 152	4.91						
						Ile 165	4.57						
	Syringic acid (8.00)	3			10	Val 29	3.99						
						Met 96	4.95						
						Ile 165	4.11						
			Cys 99	1.78		Leu 21	4.46						
			Glu 97	1.86		Val 152	5.47						
			Tyr 98	2.52		Leu 21	4.52						
						Val 29	5.11						
						Ala 42	5.05						
						Val 152	4.71						
						Ile 165	4.82						
	Vanilic acid (7.03)	1	Glu 97	2.04	7	Leu 21	4.12				1	Met 96	4.77
						Val 152	4.58						
						Val 29	5.41						
						Ala 42	4.00						
						Val 74	5.24						
						Val 152	4.94						
						Ile 165	4.33						
	IMD (7.88)	6			9	Val 29	3.93	1	Asp 103	4.06	8	Leu 21	2.81
			Glu 97	2.04		Ile 165	4.01					Leu 21	2.80
			Tyr 98	2.05		Leu 21	4.75					Cys 99	3.38
			Cys 99	2.20		Leu 21	5.41					Gln 100	3.55
			Lys 106	2.59		Val 129	5.30					Asp 103	3.56
			Gly 102	2.67		Ala 42	4.07					Asp 103	3.49
			Gly 102	2.56		Val 152	4.83					Asp 103	2.90
						Ile 165	4.42					Met 96	5.17
						Tyr 98	5.42						
